# Facile synthesis of hydantoin/1,2,4-oxadiazoline spiro-compounds via 1,3-dipolar cycloaddition of nitrile oxides to 5-iminohydantoins

**DOI:** 10.3762/bjoc.21.118

**Published:** 2025-07-31

**Authors:** Juliana V Petrova, Varvara T Tkachenko, Victor A Tafeenko, Anna S Pestretsova, Vadim S Pokrovsky, Maxim E Kukushkin, Elena K Beloglazkina

**Affiliations:** 1 Department of Chemistry, M.V. Lomonosov Moscow State University, Leninskie Gory 1-3, 119991, Moscow, Russian Federation; 2 Organic Chemistry Department, RUDN University, Miklukho-Maklaya St. 6, 117198, Moscow, Russian Federation; 3 Department of biochemistry, People’s Friendship University of Russia (RUDN University), Miklukho-Maklaya St. 6, 117198, Moscow, Russian Federation; 4 Blokhin National Medical Research Center of Oncology, Ministry of Health of the Russian Federation, 115478, Moscow, Russian Federation

**Keywords:** 1,3-dipolar cycloaddition, hydantoins, nitrile oxides, Shiff bases, spiro-compounds

## Abstract

The cycloaddition of 1,3-dipoles at C=N bonds is a relatively rare process, in contrast to the widespread cycloaddition reactions at C=C, C≡C, and C=S bonds. In this study, we present the syntheses of novel hydantoin/1,2,4-oxadiazoline spiro-compounds using a 1,3-dipolar cycloaddition of nitrile oxides to C=N bonds of 5-iminohydantoins. The efficiency of the approach was demonstrated by varying the substituents at four positions of the resulting spirocyclic molecules. Cytotoxicity of the target hydantoin/1,2,4-oxadiazolines was shown to exceed previously known spiro-compounds bearing only hydantoins or 1,2,4-oxadiazolines (IC_50_ values were 30–50 μM, HCT116 cell lines).

## Introduction

The 1,2,4-oxadiazole fragment is a common pharmacophore, and molecules containing this group exhibit a wide range of biological activities, including antitumor, anti-HIV, anti-obesity, anti-inflammatory, antidiabetic, anticancer, and antitubercular properties [1–2]. Among these molecules, bicyclic compounds with the dihydrooxadiazole connected to another cycle via the single quaternary carbon atom C^5^ demonstrated significant biological activity, greater than analogs with non-spiro-bonded cyclic fragments [3–4].

Hydantoin derivatives also exhibit a wide range of biological activities. Compounds containing the hydantoin pharmacophore group, are known for their anticancer, anti-inflammatory, antidiabetic, antimicrobial, adrenoreceptor modulating, anticonvulsant, antiplatelet, anti-HIV, and other activities [5–6]. Modifying hydantoins at the N^1^, N^3^, and C^5^ positions make it possible to achieve better pharmacological properties.

**Figure 1:**
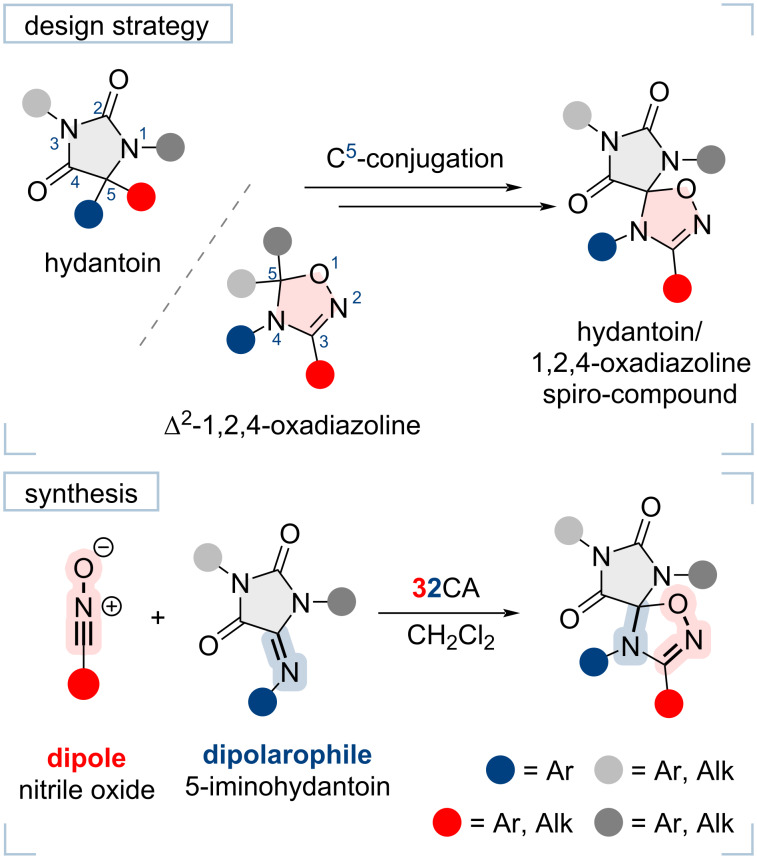
Design and synthetic strategies for the target hydantoin/1,2,4-oxadiazoline spiro-compounds.

In this paper, we firstly report the synthesis of compounds comprising two pharmacophoric fragments, hydantoin and 1,2,4-oxadiazoline, joined through the C^5^ atom of each cycle forming spiro-fused structures. We propose an efficient method for the synthesis of such spiro-compounds based on a [3 + 2]-dipolar cycloaddition (32CA) of nitrile oxides with 5-iminohydantoins (Figure 1). The proposed method involves the use of readily available starting materials, avoids chromatographic purification of the final products which are obtained in good yields. We have also tested the cytotoxicity of some synthesized spiro-hydantoin-1,2,4-oxadiazolines against the HCT116 human prostate cancer cell line, and compared it with that of compounds containing either spiro-hydantoins or spiro-oxadiazolines alone.

Although there are many examples in the literature demonstrating the ability of nitrile oxides to react with various types of double and triple bonds, most of the described reactions are related to dipolarophiles with C=C, C≡C or C=S bonds [7]. However, the [3 + 2]-cycloaddition of nitrile oxides to exocyclic C=N bonds, is a much less explored area. There are few known reactions of these dipoles with imino derivatives of oxindole [3,8–9], chrysenequinone [10], cycloheptatriene [11–12], thiazole [13], and matrine-type alkaloids [14]. The hybrid pharmacophore design is a frequently employed approach in the development of potential antitumor and other drugs [15–16]. This method involves the merging of two distinct bioactive fragments into a single molecule through a spacer. An alternative strategy of combining heterocyclic pharmacophores is to incorporate them into a spiro-jointed structure that lacks any intermediate link between the active parts [17]. Despite the fact that these strategies share numerous similarities, the molecules produced by the first method are markedly different from those produced by the latter. This is primarily due to the presence of conformational flexibility, which has a substantial impact on the compounds’ biological properties. Hybrid compounds are believed to exhibit enhanced therapeutic efficacy (lower IC_50_ values) [18] but greater toxicity to healthy cells in comparison with spiro compounds [19]. Such hybrid-designed molecules may contain a third heterocycle as a linker, spiro-joined with one of the pharmacophore moieties. In this case, another pharmacophore fragment is included in the compound as one of the substituents in the central cycle [8,20]. We believe that this approach has the potential to be a successful combination of the presented design strategies. It enables the pharmacophore fragments to be positioned relative to one another in a specific manner, as determined by the configuration of the spiro-core, while still allowing for the possibility of relative rotation to facilitate better binding to the targets. Furthermore, it appears to be as exceedingly advantageous from a synthesis perspective, as it enables the application of methods that have been developed for existing spiro systems to the new hybrid drugs.

In this work a similar modification of imidazolidine derivatives was performed for the first time for the synthesis of spiro-hydantoins. The title reactions were carried out using two alternative techniques for the generation of the reactive 1,3-dipoles. These techniques differed in the method of introducing the base necessary for generating the 1,3-dipole into the reaction medium, and included the base addition “drop by drop” or using a diffusion mixing procedure [21]. We found that the formation of spiro-compounds occurs chemo- and regioselectively for both methods. In most cases, the reactions proceeded with high yield and without the formation of significant byproducts.

## Results and Discussion

### Synthesis

**Scheme 1:**
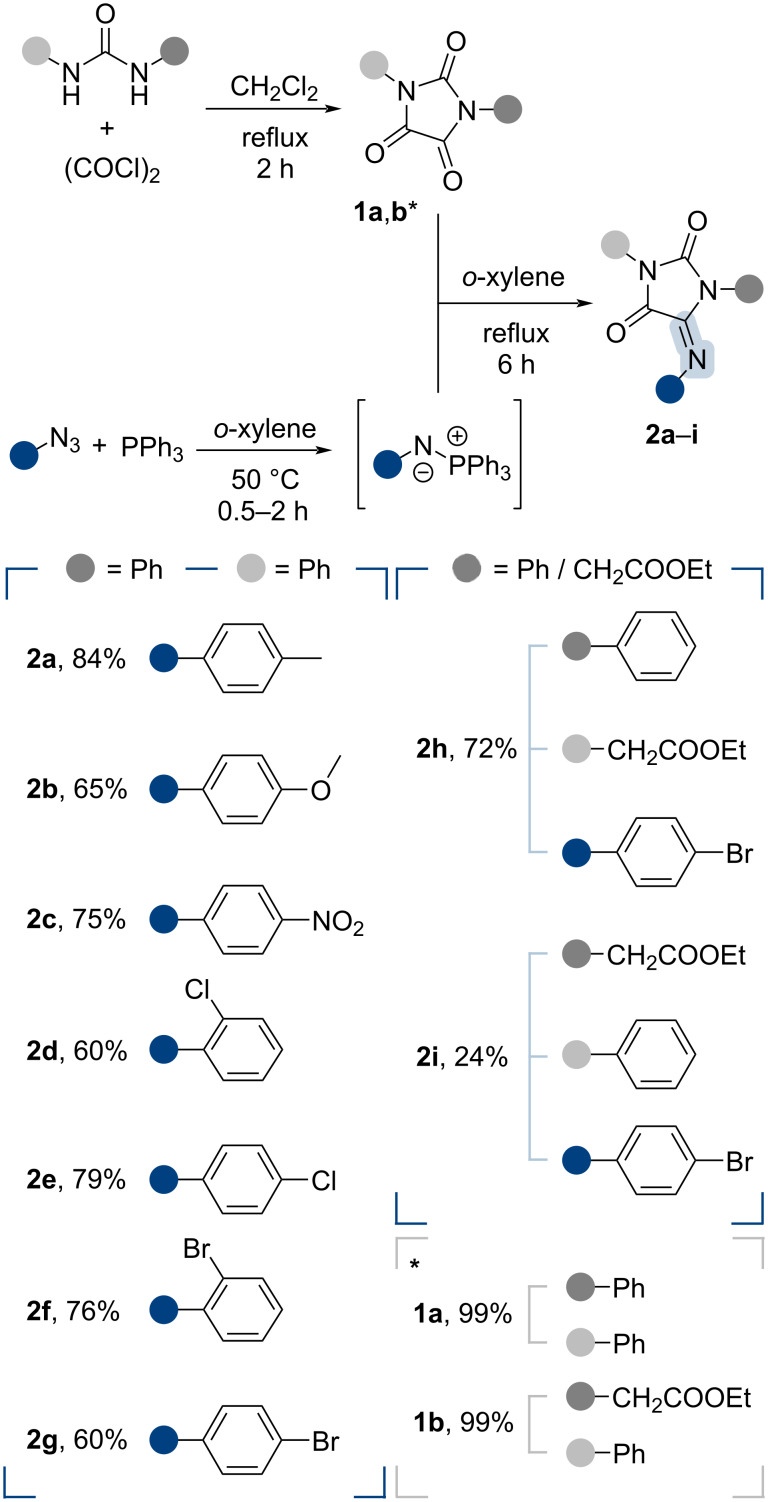
Synthesis of dipolarophiles (5-iminohydantoins **2a**–**i**).

Hydantoins **2**, containing an exocyclic C=N group, were synthesized according to the methodology described in [22] (Scheme 1). In brief, *N*,*N*'-disubstituted ureas were initially reacted with oxalyl chloride to form imidazolidinetriones **1a**,**b**, which were then added to an iminophosphorane formed in situ from an aryl azide and triphenylphosphine. As a result of the aza-Wittig reaction, 5-iminohydantoins **2a**–**i** were then used as dipolarophiles in the 32CA reactions with nitrile oxides.

**Scheme 2:**
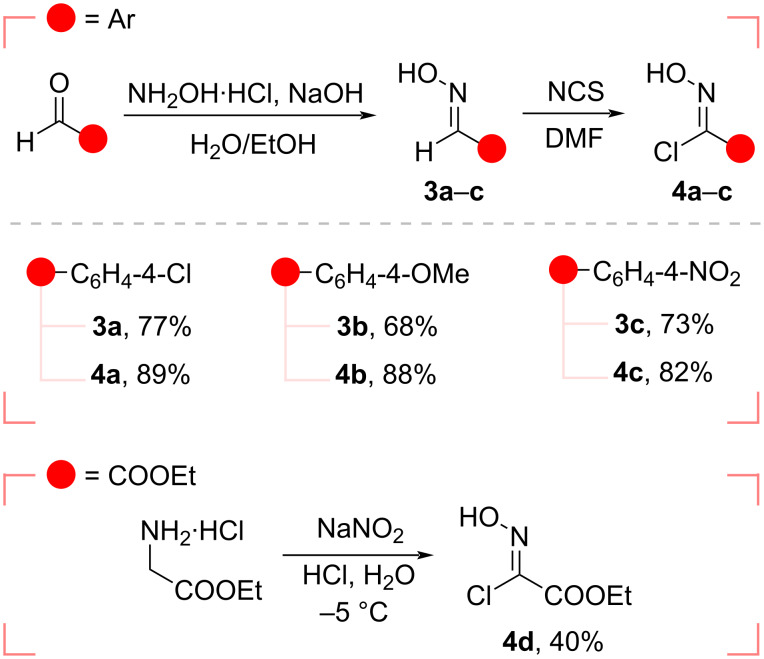
Preparation of the dipole precursors **4a**–**d**.

The nitrile oxide precursors, hydroxyimidoyl chlorides **4a**–**d** were prepared according to known methods [23–24] (Scheme 2). Conversion of benzaldehydes to the corresponding benzaldoximes **3a**–**c** was achieved through a reaction with hydroxylamine hydrochloride and a base. The dipole precursors **4a**–**c** were then prepared by the reaction of compounds **3a**–**c** with *N*-chlorosuccinimide (NCS) in dimethylformamide (DMF). The ester group-containing chloro oxime **4d** was obtained by the treatment of glycine ethyl ester hydrochloride with sodium nitrite and hydrochloric acid [25] and used thereafter as carbethoxyformonitrile oxide (CEFNO) precursor.

**Scheme 3:**
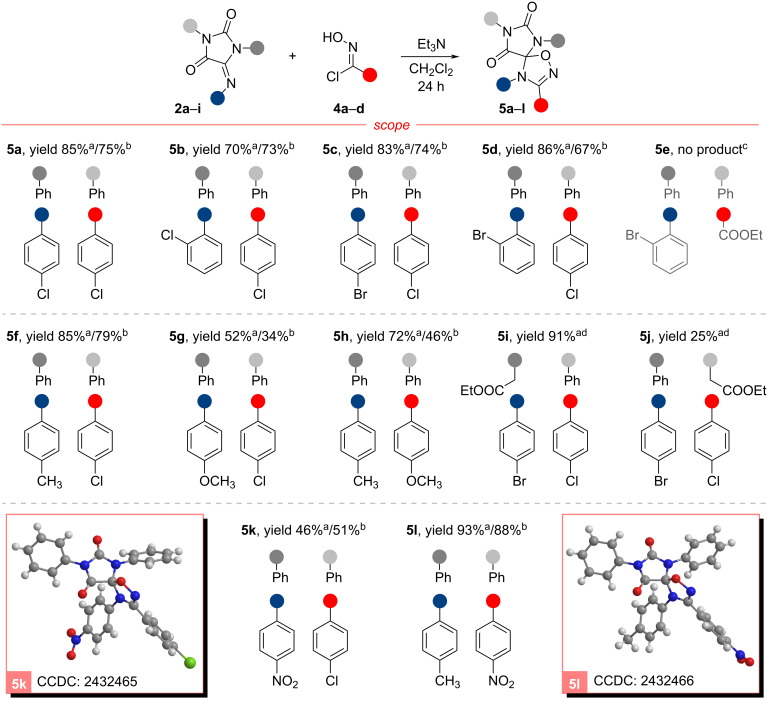
32CA reactions of nitrile oxides with 5-iminohydantoins (synthesis of spiro-compounds **5a**–**l**). Isolated yields are shown. Unless otherwise noted dipolarophile **2** (0.15 mmol) and chloro oxime **4** (0.165 mmol) were used, and the product **5** was isolated via Et_2_O trituration. ^a^Triethylamine dropwise addition (1.2 equiv, 0.06 M CH_2_Cl_2_ solution, Ar, 0–5 °C); ^b^diffusion mixing technique (for details see Supporting Information File 1), ^c^using diffusion mixing *N*-(2-bromophenyl)formamide (**6**) was obtained with 80% yield; ^d^product was isolated using column chromatography.

The nitrile oxides were generated in situ by the action of a base (Et_3_N) to compounds **4a**–**d** in the dipolarophile-containing solution to prevent high concentrations of dipole molecules and to minimize the inevitable competition between the 32CA reaction and the isomerization of nitrile oxides to isocyanates or their dimerization [26–27]. Since isomerization is believed to be the primary direction of molecule degradation only for bulky *o*,*o*'-disubstituted aromatic dipoles [28–30], the main efforts in developing a methodology were focused on suppressing dimerization processes. It is worth noting that, in some cases, due to the high reactivity of nitrile oxides towards certain types of dipolarophiles, such as monosubstituted or activated by electron-acceptor groups olefins [31], no additional attention to this is required. However, when introducing obstructed dipolarophiles into the 32CA, it is reasonable to utilize conditions that minimize the side reactions of the dipole. This could include lowering the temperature [32] and slowly adding a base [33]. In this work we have tested two alternative techniques for the dipolar cycloaddition. In the first method, a triethylamine solution was slowly added dropwise to the reaction mixture at 0 °C under an inert gas atmosphere to avoid moisture from the air (“classical” method). In another method, the base was inserted via diffusion of NEt_3_ into a solution containing the dipolarophile and dipole precursor (see Figure S1 in Supporting Information File 1 for a schematic reaction setup) [21]. This method, previously tested in other reactions of nitrile oxides [21] and nitrile imines [22], was chosen as a reliable technique for the slow and consistent generation of the reactive dipole. In all cases the dipole precursor **4** was used in small excess (1.1 equiv) over the dipolarophile **2** (1 equiv). Using both methods, a series of products **5a**–**l** containing spiro-conjugated fragments of hydantoin and 1,2,4-oxadiazole was obtained (Scheme 3).

In most cases, the use of the “classical” drop-by-drop method gave compounds **5a**–**l** in yields near or superior to those obtained by diffusion mixing. However, there were three exceptions (**5d**, **5g** and **5h**) where we observed a significant difference in the results. The most striking example was compound **5h**, which exhibited a yield decrease of more than a third when the diffusion mixing technique was employed. The substantial impact of the reaction conditions can be attributed to the stability of the dipole formed. The presence of mesomeric donor substituent (such as 4-OMe group) has been shown to render nitrile oxides more susceptible to dimerization in comparison to other dipoles [27,34]. The most dramatic discrepancy in spiro-compound yields was observed during the reaction with this nitrile oxide, suggesting that classical conditions (low temperature and inert atmosphere) may prove more fruitful in preventing the degradation of the dipole. The influence of the concentration of the generated nitrile oxide seems to be less significant.

It was found that the low solubility of products containing aromatic substituents in non-polar solvents allows for their isolation by washing the crude reaction mixture with diethyl ether to remove organic impurities and then with water to eliminate triethylamine hydrochloride. This method has proven to be simple and effective in most cases, with the exception of compound **5g**. The moderate solubility of this compound in diethyl ether led to partial precipitation from the reaction mixture. It is worth noting that chromatographic purification is also possible for compounds **5a**–**l**. However, in most cases, this method is more laborious due to the similarity in the retention factor values of the initial imines **2** and the reaction products. In this way, products **5i** and **5j** with good solubility in diethyl ester were isolated.

A comparative analysis of compounds **5a**–**l** yields revealed that in the absence of aromatic substituents with strong electron-donating or electron-accepting properties, the reaction occurred with near-complete conversion of the initial dipolarophile and high yields of the products (as, for example, **5a**–**d** and **5f**). The best result, however, was observed for the spiro-compound **5l**, which contains a *p*-nitro group in the aromatic core of the nitrile oxide fragment. This compound was obtained in excellent yield of 93% and 88% using the dropwise and diffusion mixing technique, respectively. This result may be explained by the optimal congruence of the electronic properties of the substituents in the dipole and dipolarophile, as well as very low solubility of the product **5l** in diethyl ether.

An unexpected result was obtained during the 1,3-dipolar cycloaddition reaction between **2f** and chloro oxime **4d**. In contrast to reactions involving benzonitrile oxides, in this case the dropwise addition method of triethylamine resulted in complete inactivity of the dipole towards the dipolarophile **2f**, which was quantitatively recovered from the reaction mixture. The low reactivity of CEFNO can be explained by considering nitrile oxides in terms of frontier molecular orbital (FMO) theory. Nitrile oxides are considered to be "type II" dipoles capable of reacting both through the interaction of HOMO_dipole_–LUMO_dipolarophile_ and LUMO_dipole_–HOMO_dipolarophile_ [35]. According to the results presented in [36], reactions with benzonitrile oxide can satisfy both normal and inverse-electron demands, depending on the electronic properties of substituents in the aromatic fragments of the dipole and dipolarophile. Our result may indicate a predominant overlap of HOMO_dipole_ and LUMO_dipolarophile_ (inverse-electron demands) in the reactions of the imine **2f**. In this case, the strong electron-withdrawing properties of the carboxyethyl group in CEFNO can significantly inhibit the reaction with **2f**, lowering the energy of the HOMO_dipole_ involved in the interaction. Instead, the reaction can proceed towards dipole dimerization [37], which is generally much easier for nitrile oxides that are not stabilized by an aromatic group [28]. By performing the diffusion mixing technique, we were also unable to detect the cycloaddition product. Instead, after 3 weeks of reaction, exceptionally *N*-(2-bromophenyl)formamide (**6**) was isolated with 80% yield. Apparently, it is the product of decomposition of the initial imine **2f**. No other substances could be identified in this case.

In all cases, the 32CA reactions occurred regiospecifically, forming a 1,2,4-oxadiazoline ring, which is consistent with the results of the reactions for similar substrates [36,38]. The regioselectivity of the cycloaddition reaction between nitrile oxides and C=N bonds can be rationalized by considering the relative electronegativities of the terminal elements and the distribution of electron density in the frontier orbitals of the reagents [35]. The electrons are preferentially located on the more electronegative oxygen or nitrogen atoms, both in the HOMO of dipole and dipolarophile [36,39]. In contrast, the carbon atoms have a significantly higher orbital coefficient than the heteroatoms, both in the LUMO_dipolarophile_ and the LUMO_dipole_. Thus, nitrile oxide tends to react with an imine to form two new carbon–heteroatom bonds in a 1,2,4-oxadiazoline ring through both variants of orbital overlap. It should be noted that, in this case, NMR spectroscopy is not applicable to assign the product structure due to the presence of only quaternary carbon atoms in the tetrasubstituted 1,2,4-oxadiazolines [40]. Therefore, single crystal X-ray diffraction analysis is typically used to confirm the structure, as we have applied for the hydantoin/1,2,4-oxadiazoline spiro-compounds **5k** (CCDC 2432465) and **5l** (CCDC 2432466) (for details see Supporting Information File 1).

The effect of the substituents on the N^1^ and N^3^ nitrogen atoms in the hydantoin core was studied in the reactions of hydroxyimidoyl chloride **4a** with dipolarophiles **2g**, **2h**, and **2i**, resulting in the products **5c** (83% yield), **5i** (91%), and **5j** (25%), respectively (Scheme 3). The replacement of a phenyl (**2g**) with an alkyl substituent at the N^1^ nitrogen atom (**2i**) led to a sharp decrease in the yield of the cycloaddition product **5j**, whereas the presence of the N^3^-CH_2_COOEt (**2h**) fragment slightly enhanced the yield of **5i** compared to the bis-phenyl substituted dipolarophile **2g**. The low yield observed for product **5j** may be accounted for by a greater involvement of the N^1^ electron pair in conjugation with the imide fragment of the dipolarophile and its deactivation in the reaction with nitrile oxide. This pattern is consistent with the one previously observed when studying the reactions of these dipolarophiles with nitrile imines [22].

**Scheme 4:**
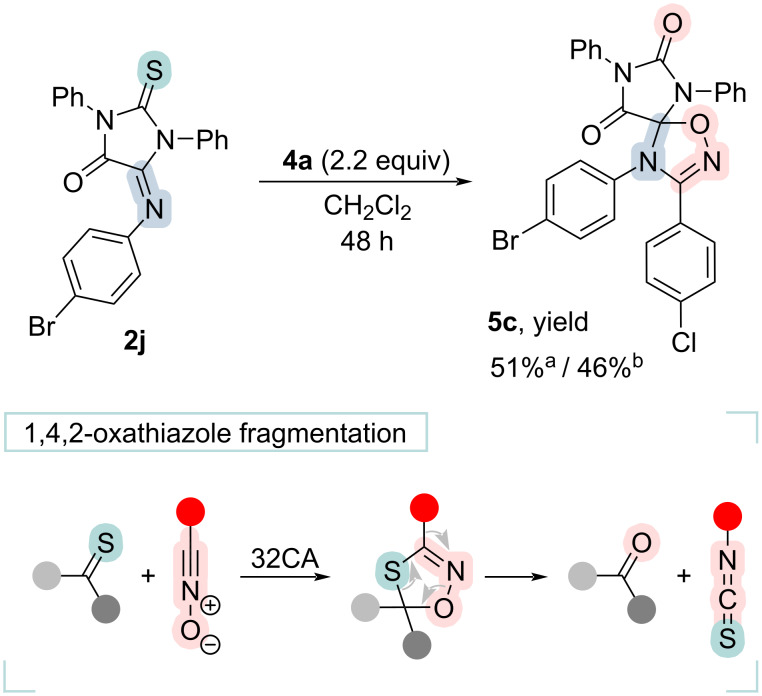
Cycloaddition of nitrile oxide to 5-iminothiohydantoin **2j**. ^a^Triethylamine dropwise addition (2.4 equiv, 0.06 M CH_2_Cl_2_ solution, Ar, 0–5 °C); ^b^diffusion mixing technique.

It is known that nitrile oxides are capable of reacting with various multiple carbon–heteroatom bonds [28]. In contrast to the products of nitrile oxide cycloaddition to imino groups, 1,4,2-oxathiazoles, formed by addition to C=S bonds, are unstable and undergo decomposition into isothiocyanate and a carbonyl-containing compound [38]; the stability of these compounds is contingent on the nature of the substituents bonded to the thiocarbonyl group, which reacts with nitrile oxide [41]. It has been demonstrated [7] that 1,4,2-oxathiazoles derived from thiourea are among the least stable compounds, gradually decomposing even at room temperature. Taking these into account, we have synthesized compound **5c** using an alternative route starting from the chloro oxime **4a** and 5-iminothiohydantoin **2j** (Scheme 4), obtained similarly to other dipolarophiles [22]. The total yield of the product **5c** in this case turned out to be comparable to that obtained for all stages of the initial scheme (synthesis of **5c** from **2g**, Scheme 3). The yield of the desulfurized product was moderate both with triethylamine dropwise addition (51%) and with diffusion mixing technique (46%).

**Figure 2:**
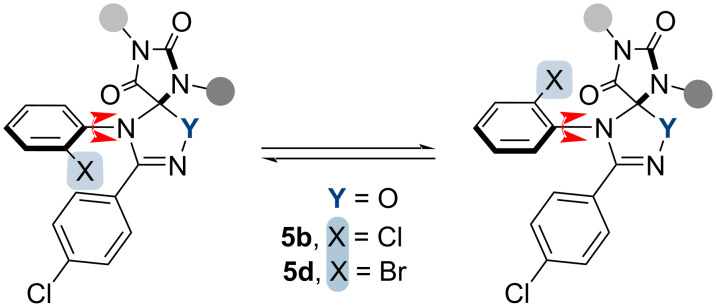
Atropoisomerism of *ortho*-substituted spiro-compounds **5b** and **5d**.

Interestingly, for spiro-compounds **5b** and **5d** containing an *ortho*-substituted aromatic group, we have observed atropoisomerism (Figure 2) what is manifested by the appearance of a second set of signals in their NMR spectra. As the previously studied similar structures (Y = N–R) [22], products **5b** and **5d** (Y = O) were formed through the addition of a dipole molecule, resulting in the formation of two new carbon–heteroatom bonds and the formation of a five-membered ring. Thus, the aryl attached to the nitrogen atom from the dipolarophile fragment is brought closer to the substituent from the C-end of the dipole. This proximity in the presence of *ortho*-substituents in the aromatic core hinders rotation around the C–N bond.

### Cytotoxicity

The cytotoxic properties of hydantoin/1,2,4-oxadiazoline spiro-compounds were investigated using the MTT [42] assay on human colorectal carcinoma cell line HCT116.

**Figure 3:**
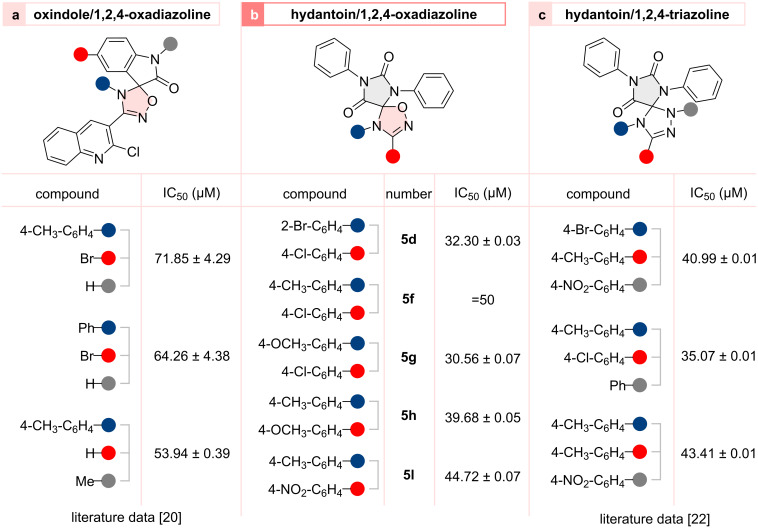
Cytotoxicity investigation of hydantoin/1,2,4-oxadiazolines **5** (MTT test, HCT116 cell line) and selected examples of oxindole/1,2,4-oxadiazole [20] and hydantoin/1,2,4-triazoline [22] spiro-compounds.

To evaluate the cytotoxicity of the compounds in vitro, cells were placed in 96-well culture plates at a concentration of 4–7 × 10^3^ cells/mL and incubated at 37 °C for 24 hours. The cells were counted after treatment with trypan blue solution (0.4%) in a Goryaev chamber. Then, after incubation at 37 °C for 72 h, the cells were exposed to various concentrations of the studied compounds in two-fold serial (50–100 μM) dilutions. Only DMSO + PBS (phosphate-buffered saline) was used as a control, since the studied compounds were soluble only in DMSO. Cell viability was measured using the standard MTT test [42]. Absorbance was measured at 540 nm using a Multiskan™ FC microplate reader and Skanlt 6.1 RE software for a microplate reader, both from Thermo Scientific (Waltham, MA, USA). In vitro experiments were performed in triplicate. Graphpad prism version 9.0 was used to determine the IC_50_. IC_50_ data are presented as mean ± standard deviation (SD) (Figure 3b) [20,22].

Notwithstanding the moderate cytotoxicity exhibited by the obtained compounds, they demonstrated a slight improvement over those previously obtained for the hydantoin/1,2,4-triazoline spiro-compounds [22] (Figure 3c, IC_50_ ≈ 35–45 µM) and were more prominent than oxindolo/1,2,4-oxadiazoles (Figure 3a, IC_50_ ≈ 50–5 µM) tested on this cell line in the reference [20].

Two of the five compounds studied in this work (**5d** and **5g**) demonstrated IC_50_ values of about 30 µM. The data obtained indicates that products **5** exhibit more pronounced cytotoxic properties when they contain a *p*-methoxy group in the dipole fragment (Figure 3b, red ring) than 4-NO_2_ or 4-Cl substituents (IC_50_ of **5h** was lower than **5l** and **5f**). The presence of a methoxy group in the imine aromatic core (blue ring) also had a significant impact on cytotoxicity. This can be concluded from the results obtained for compounds **5f** and **5g**: the substitution of -CH_3_ to -OCH_3_ led to a decrease in IC_50_ by at least 20 µM and turned out to be comparable to that obtained for **5d**.

## Conclusion

In this study, we firstly investigated the 1,3-dipolar cycloaddition reactions of nitrile oxides to 5-iminohydantoins at their exocyclic C=N bond. A convenient preparative synthesis of hydantoin/1,2,4-oxadiazoline spiro-compounds using two different methods for introducing a tertiary amine into the reaction mixture containing the dipolarophile and precursor of the 1,3-dipole (chloro oxime), either by dropwise addition or by diffusion mixing. By varying the substituents on N^1^, N^3^, and the imino group of the dipolarophile as well as the dipole structure (aryl and alkyl), we determined the synthetic potential of the method and identified the most favorable electronic and steric combinations of substituents that affect the course of the studied 32CA reactions. The cytotoxicity of the obtained compounds was evaluated using the HCT116 cell line. It has been demonstrated that the combination of the hydantoin and 1,2,4-oxadiazoline moieties in a single spiro-fused system leads to increased cytotoxic activity compared to other spiro-derivatives of hydantoin and 1,2,4-oxadiazole.

## Experimental

### General procedure for the synthesis of the spirocyclic products

**Triethylamine dropwise addition:** A solution of dipolarophile **2** (1 equiv, 0.150 mmol) and *N*-hydroxybenzimidoyl chloride **4** (1.1 equiv, 0.165 mmol) in 4.5 mL of DCM was added to a 25 mL round-bottomed flask equipped with a magnetic stirring bar and a dripping funnel. After a solution of Et_3_N (1.2 equiv, 0.180 mmol, 25 µL) in DCM (3 mL) had been added to the funnel, the system was filled with argon and placed in an ice bath. A few minutes later, the Et_3_N solution was added dropwise to the mixture in the flask, while it was stirred. After that, the reaction mixture was left to stir in the ice bath for 24 hours, allowing it to slowly reach room temperature.

**Diffusion mixing technique:** A small vial (15 mL volume, diameter 1.3 cm) equipped with a magnetic stirring bar was charged with a mixture of dipolarophile **2** (1 equiv, 0.150 mmol) and hydroxyimidoyl chloride **4** (1.1 equiv, 0.165 mol) in 4.5 mL of DCM and then placed in larger vial (50 mL volume, diameter 3.5 cm) containing TEA (35.85 mmol, 5 mL). The outer vial was tightly closed with a lid and the reaction mixture was stirred at room temperature for 24 h.

In both cases, after the reaction time had expired, the solvent was removed in vacuo. The residue was washed with 2–3 small portions (0.5–1 mL) of Et_2_O and water giving a solid precipitate of the product (compounds **5a**–**d, 5f**–**h** and **5k**–**l**). Compounds **5i** and **5j** were isolated from the reaction mixtures by column chromatography on silica gel using DCM as eluent.

## Supporting Information

File 1 Detailed experimental procedures, characterization data and X-ray crystallographic details.

## Data Availability

All data that supports the findings of this study is available in the published article and/or the supporting information of this article.
