# Supplementary material for: Facile synthesis of hydantoin/1,2,4-oxadiazoline spiro-compounds via 1,3-dipolar cycloaddition of nitrile oxides to 5-iminohydantoins
Source: Beilstein J Org Chem. 2025 Jul 31;21:1552–60. doi: 10.3762/bjoc.21.118 (PMC12320734; doi:10.3762/bjoc.21.118)
Supplement: File 1 — Detailed experimental procedures, characterization data and X-ray crystallographic details. [file Beilstein_J_Org_Chem-21-1552-s001.pdf]

BEILSTEIN JOURNAL OF ORGANIC CHEMISTRY

Supporting Information

for

Facile synthesis of hydantoin/1,2,4-oxadiazoline spiro-compounds via 1,3-dipolar cycloaddition of nitrile oxides to 5-iminohydantoins

Juliana V. Petrova, Varvara T. Tkachenko, Victor A. Tafeenko, Anna S. Pestretsova, Vadim S. Pokrovsky, Maxim E. Kukushkin and Elena K. Beloglazkina

Beilstein J. Org. Chem. **2025,** *21,* 1552–1560. doi:10.3762/bjoc.21.118

Detailed experimental procedures, characterization data and X-ray crystallographic details

License and Terms: This is a supporting information file under the terms of the Creative Commons Attribution License (https://creativecommons.org/licenses/by/4.0). Please note that the reuse, redistribution and reproduction in particular requires that the author(s) and source are credited and that individual graphics may be subject to special legal provisions.
The license is subject to the *Beilstein Journal of Organic Chemistry* terms and conditions: (https://www.beilstein-journals.org/bjoc/terms)

Table of contents

List of abbreviations S2

Materials and methods S2

Preparation of hydroximidoyl chlorides (nitrile oxide precursors) S4

General procedure for the synthesis of the spirocyclic products S6

X-ray crystallographic details S11

Cytotoxicity evaluation S13

References S14

^{1}H and ^{13}C spectra for compounds **5a–l** S15

List of abbreviations

DCM – dichloromethane (CH_2Cl_2)

DMF – *N*,*N*-dimethylformamide

DMSO – dimethyl sulfoxide

DMT – diffusion mixing technique

MTT – 3-(4,5-dimethylthiazol-2-yl)-2,5-diphenyltetrazolium bromide

NCS – *N*-chlorosuccinimide

PBS – phosphate-buffered saline

SD – standard deviation

TDA - triethylamine dropwise addition

TLC – thin-layer chromatography

Materials and methods

Reagents were purchased from commercial sources and used without further purification. All solvents were purified and dehydrated as described[1]. Reactions were monitored by TLC on silica plates with a fluorescent indicator (254 nm) and visualized with a UV lamp.

Nuclear magnetic experiments were recorded using Bruker Avance (Bruker Optik GmbH, Ettlingen, Germany) and Agilent 400-MR (Agilent Technologies, Santa Clara, CA, US) spectrometers operating at 400 MHz for ^{1}H and 101 MHz for ^{13}C nuclei. Chemical shifts were reported as δ units (ppm) relative to the residual peak of solvents (ref: $CDCl_3$/DMSO-d_6, ^{1}H: 7.26/2.50 ppm; ^{13}C: 77.16/39.52 ppm) and coupling constants (J) in Hz.

Electrospray ionization high-resolution mass spectra were recorded in the positive ion mode on a TripleTOF 5600+ quadrupole time-of-flight mass spectrometer (ABSciex, Concord, Vaughan, ON, Canada) equipped with a DuoSpray ion source. The following MS parameters were applied: capillary voltage 5.5 kV; nebulizing and curtain gas pressures – 15 and 25 psi, respectively; ion source temperature – ambient; declustering potential 20 V; *m*/*z* range 100–1200. Elemental compositions of the detected ions were determined based on accurate masses and isotopic distributions using Formula Finder software (ABSciex, Concord, ON, Canada). The maximum allowed deviation of the experimental molecular mass from the calculated one was 5 ppm.

The X-ray data were collected via STOE diffractometer Pilatus100K detector (DECTRIS AG, Baden, Switzerland), Cu Kα (1.54086Å) radiation, rotation method mode, temperature 295(2) K. STOE X-AREA software was used for cell refinement and data reduction (full-matrix least-squares on F2 refinement method). Data collection and image processing were performed with X-Area 1.67 (STOE & Cie GmbH, Darmstadt, Germany, 2013). Intensity data were scaled with LANA (part of X-Area) in order to minimize differences in intensities of symmetry-equivalent reflections (multi-scan method). CCDC 2432465 (compound **5k**), 2432466 (**5l**) contain the supplementary crystallographic data for this paper. The data can be obtained free of charge from the Cambridge Crystallographic Data Centre via www.ccdc.cam.ac.uk/data_request/cif website.

The MTT assay was used to assess the cytotoxicity of the synthesized compounds. Sigma-Aldrich (Schnelldorf, Germany) provided 3-(4,5-dimethylthiazol-2-yl)-2,5-diphenyltetrazolium bromide (MTT). Trypan blue, phosphate-buffered saline (PBS), and dimethyl sulfoxide (DMSO) were

purchased from PanEco (Moscow, Russia). Fetal bovine serum was obtained from HyClone (Logan, UT, USA), along with flasks and plates purchased from Nunc (Moscow, Russia). Cell line: human colon adenocarcinoma cell line (HCT116) was purchased from ATCC (Manassas, VA, USA). Cancer cells were routinely grown in RPMI 1640 culture medium, supplemented with 10% fetal bovine serum, glutamine, and 100 U/mL penicillin. HCT116 cell line was grown in flasks in RPMI 1640 fresh culture medium with supplements at 37 °C and 5% CO_2. Cells were grown as monolayer cultures, and the cells in the exponential growth phase were trypsinized and suspended in supplemented RPMI 1640 medium.

Compounds **1a**–**c** and **2a**–**j** were obtained as described previously [1,2]. Their characterization data agreed with reported analysis.

Preparation of hydroximidoyl chlorides (nitrile oxide precursors)

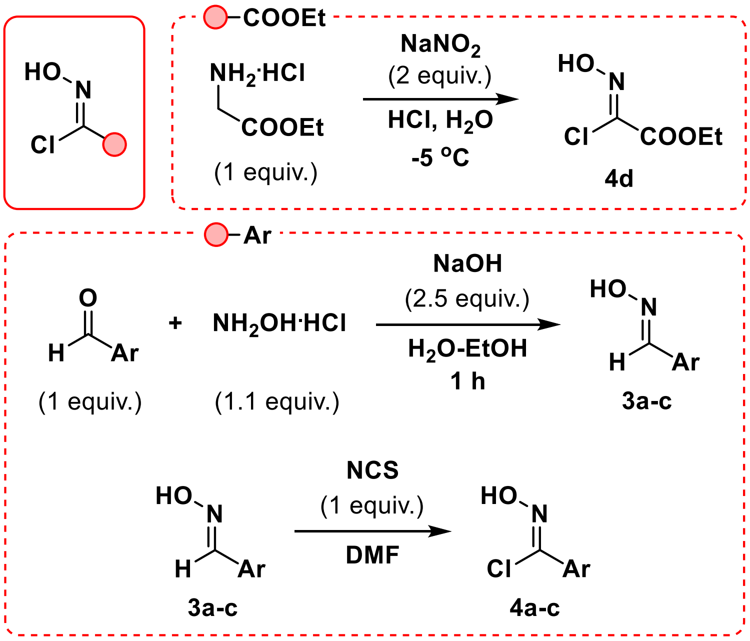

Benzohydroximidoyl chlorides **4a–c** were synthesized according to the following two-step procedure: benzaldehydes were firstly converted to corresponding oximes **3a–c** via reaction with hydroxylamine hydrochlorides and sodium hydroxide and then to hydroximidoyl chlorides **4** by treatment with *N*-chlorosuccinimide [3].

Ethyl chlorooximidoacetate (**4d**) was obtained by treatment of glycine ethyl ester hydrochloride with hydrochloric acid and sodium nitrite [4].

General procedure for benzaldoximes 3a–c preparation [3]

Benzaldehyde (1 equiv) was added to a water/ethanol mixture 1:1 (v/v, 1 mL/mmol). The reaction solution was placed in a cool water bath, and charged with hydroxylamine hydrochloride (1.1 equiv) and 50% water solution of NaOH (2.5 equiv). After stirring for 1 h at room temperature, the mixture was washed with Et_2O (1 mL/mmol), acidified to pH 6 with concentrated HCl, and extracted with DCM (2 × 1 mL/mmol). The latter two organic layers were combined and dried over Na_2SO_4. The solvent was removed in vacuo, giving the crude products **3a–c**.

4-Chlorobenzaldehyde oxime (**3a**) [5]

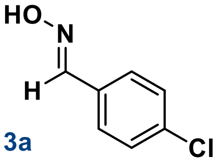

3a

Compound **3a** was obtained from 4-chlorobenzaldehyde (0.70 g, 5.0 mmol), hydroxylamine hydrochloride (0.38 g, 5.5 mmol) and NaOH (0.50 g, 12.5 mmol) as white crystalline solid (0.61 g, 77% yield).

^{1}H NMR (400 MHz, DMSO-d_6) δ 11.39 (s, 1H, N-OH), 8.15 (s, 1H, N=C-H), 7.61-7.59 (m, 2H, Ar), 7.46-7.44 (m, 2H, Ar).

4-Methoxybenzaldehyde oxime (**3b**) [6]

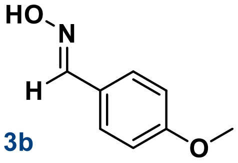

3b

Compound **3b** was obtained from 4-methoxybenzaldehyde (0.68 g, 5.0 mmol), hydroxylamine hydrochloride (0.38 g, 5.5 mmol) and NaOH (0.50 g, 12.5 mmol) as beige crystallizing oil (0.51 g, 68% yield).

^{1}H NMR (400 MHz, $CDCl_3$) δ 8.10 (s, 1H, N=C-H), 7.54 – 7.49 (m, 2H, Ar), 6.94 – 6.89 (m, 2H, Ar), 3.84 (s, 3H, OCH_3).

4-Nitrobenzaldehyde oxime (**3c**) [6]

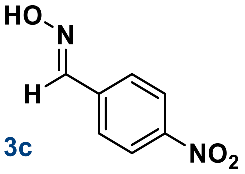

3c

Compound **3c** was obtained from 4-nitrobenzaldehyde (0.76 g, 5.0 mmol), hydroxylamine hydrochloride (0.38 g, 5.5 mmol) and NaOH (0.50 g, 12.5 mmol) as beige crystallizing oil (0.61 g, 73% yield).

^{1}H NMR (400 MHz, $CDCl_3$) δ 8.28 (s, 1H, N-OH), 8.26 – 8.22 (m, 2H, Ar), 8.20 (s, 1H, N=C-H), 7.78 – 7.72 (m, 2H, Ar).

General procedure for *N*-hydroxybenzoimidoyl chlorides 4a–c preparation [3]

A stirring solution of benzaldehyde oxime **3a–c** (2.5 mmol) in DMF (5 mL) was charged with NCS (67 mg, 0.5 mmol) and gently heated to 50 °C. After the reaction was initiated, the remaining NCS (267 mg, 2 mmol) was added and the mixture was allowed to stir overnight at room temperature. Then the mixture was diluted with ice-cold water (20 mL) and extracted with Et_2O (2 × 20 mL). The combined organic layers were washed with water (3 × 20 mL) and dried over Na_2SO_4. The solvent was removed in vacuo, giving the crude product of *N*-hydroxybenzimidoyl chlorides **4a–c**.

4-Chloro-*N*-hydroxybenzimidoyl chloride (**4a**) [3]

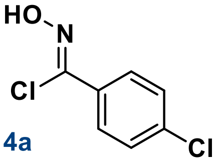

4a

Compound **4a** was obtained from oxime **3a** (0.39 g, 2.5 mmol) and NCS (0.33 g, 2.5 mmol) as beige powder (0.42 g, 89% yield).

^{1}H NMR (400 MHz, $CDCl_3$) δ 7.87 (s, 1H, N-OH), 7.82 - 7.75 (m, 2H, Ar), 7.41 - 7.36 (m, 2H, Ar).

N-Hydroxy-4-methoxybenzimidoyl chloride (**4b**) [7]

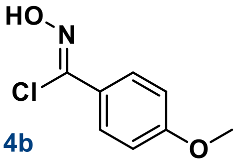

4b

Compound **4b** was obtained from oxime **3b** (0.38 g, 2.5 mmol) and NCS (0.33 g, 2.5 mmol) as beige powder (0.41 g, 88% yield).

^{1}H NMR (400 MHz, $CDCl_3$) δ 7.81 – 7.72 (m, 3H, $2H_{Ar}$+N-OH), 6.96 – 6.88 (m, 2H, Ar), 3.85 (s, 3H, OCH_3).

N-Hydroxy-4-nitrobenzimidoyl chloride (**4c**) [7]

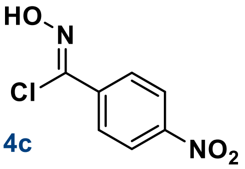

4c

Compound **4c** was obtained from oxime **3c** (0.42 g, 2.5 mmol) and NCS (0.33 g, 2.5 mmol) as beige powder (0.41 g, 82% yield).

^{1}H NMR (400 MHz, $CDCl_3$) δ 8.27 (d, *J* = 9.1 Hz, 2H, Ar), 8.08 – 8.01 (m, 3H, $2H_{Ar}$+N-OH).

Synthesis of ethyl 2-chloro-2-(hydroxyimino)acetate (4d) [4]

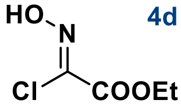

4d

Concentrated HCl (4.15 mL) was added to a stirring solution of glycine ethyl ester hydrochloride (6.98 g, 50 mmol) in 9.5 mL of water at −5 °C. Then a solution of sodium nitrite (3.45 g, 50 mmol) in 5 mL of water was added dropwise. After that the same amount of HCl and sodium nitrite was added. The reaction mixture was stirred for 3 hours, and extracted with Et_2O (2 × 25 mL). The combined organic phase was dried over Na_2SO_4, and the solvent was evaporated. Ethyl 2-chloro-2-(hydroxyimino)acetate (**4d**) was obtained as crystallizing oil (3 g, 40% yield).

^{1}H NMR (400 MHz, $CDCl_3$) δ 10.00 (s, 1H, N-OH), 4.40 (q, *J* = 7.1 Hz, 2H, CH_2), 1.39 (t, *J* = 7.1 Hz, 3H, CH_3).

General procedure for the synthesis of the spirocyclic products

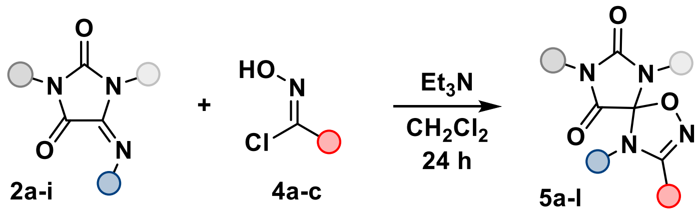

Triethylamine dropwise addition (TDA): A solution of dipolarophile **2** (1 equiv, 0.150 mmol) and *N*-hydroxybenzimidoyl chloride **4** (1.1 equiv, 0.165 mmol) in 4.5 mL of DCM was added to a 25 mL round-bottomed flask equipped with a magnetic stirring bar and a dripping funnel. After a solution of Et_3N (1.2 equiv, 0.180 mmol, 25 µL) in DCM (3 mL) had been added to the funnel, the system was filled with argon and placed in an ice bath. A few minutes later, the Et_3N solution was added dropwise to the mixture in the flask, while it was stirred. After that, the reaction mixture was left to stir in an ice bath for 24 hours, allowing it to slowly reach room temperature.

Diffusion mixing technique (DMT) [8]: Small vial (15 mL volume, diameter 1.3 cm) equipped with a magnetic stirring bar was charged with a mixture of dipolarophile **2** (1 equiv, 0.150 mmol) and hydroximoyl chloride **4** (1.1 equiv, 0.165 mol) in 4.5 mL of DCM and then placed in larger vial (50 mL volume, diameter 3.5 cm) containing Et_3N (35.85 mmol, 5 mL). The outer vial was tightly closed with a lid and the reaction mixture was stirred at room temperature for 24 h.

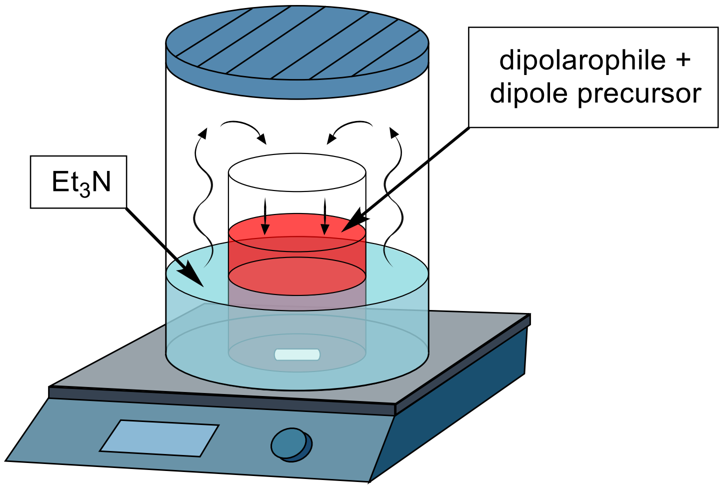

Figure S1. Equipment for carrying out reactions using diffusion mixing technique.

In both cases, after the reaction time had expired, the solvent was removed in vacuo. The residue was washed with 2–3 small portions (0.5–1 mL) of Et_2O and water giving a solid precipitate of the product (compounds **5a–d, 5f–h** and **5k–l**). Compounds **5i** and **5j** were isolated from the reaction mixtures by column chromatography on silica gel using DCM as eluent.

3,4-Bis(4-chlorophenyl)-6,8-diphenyl-1-oxa-2,4,6,8-tetraazaspiro[4.4]non-2-ene-7,9-dione (**5a**)

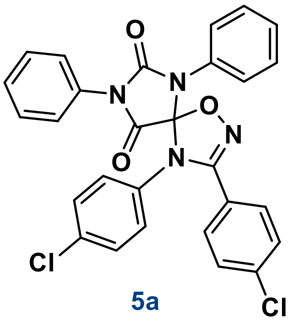

5a

Compound **5a** was obtained from dipolarophile **2a** (56 mg, 0.150 mmol), (*Z*)-4-chloro-*N*-hydroxybenzimidoyl chloride (**4a**, 31 mg, 0.165 mmol) as light beige solid (*TDA*: 67 mg, 85% yield; *DMT*: 59 mg, 75% yield).

^{1}H NMR (400 MHz, DMSO-d_6) δ 7.65 – 7.60 (m, 2H, Ar), 7.55 – 7.40 (m, 10H, Ar), 7.34 – 7.30 (m, 2H, Ar), 7.30 – 7.25 (m, 2H, Ar), 7.09 – 7.02 (m, 2H, Ar).

^{13}C{^{1}H} NMR (101 MHz, DMSO-d_6) δ 163.5, 153.2, 151.1, 136.4, 133.7, 132.3, 130.3, 130.0, 129.8, 129.5, 129.4, 129.3, 129.2, 128.7, 126.9, 126.9, 121.5, 104.7.

HRMS (ESI) calcd for $C_{28}H_{18}Cl_2N_4O_3$ $[M+H]^+$ *m/z* 529.0829, found *m/z* 529.0840.

4-(2-Chlorophenyl)-3-(4-chlorophenyl)-6,8-diphenyl-1-oxa-2,4,6,8-tetraazaspiro[4.4]non-2-ene-7,9-dione (**5b**)

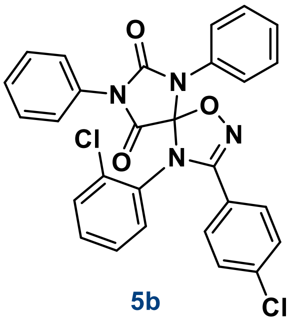

5b

Compound **5b** was obtained from dipolarophile **2b** (56 mg, 0.150 mmol), (*Z*)-4-chloro-*N*-hydroxybenzimidoyl chloride (**4a**, 31 mg, 0.165 mmol) as yellowish solid (*TDA*: 55 mg, 70% yield; *DMT*: 58 mg, 73% yield).

Mixture of isomers I and II (2:1 in DMSO-d_6):

^{1}H NMR (400 MHz, DMSO-d_6) δ 7.70 (d, *J* = 6.4 Hz, 2H$^{\mathbf{I}}$, Ar), 7.60 (d, *J* = 7.6 Hz, 2H$^{\mathbf{II}}$, Ar), 7.55 – 7.31 (m, 12H$^{\mathbf{I}}$+14H$^{\mathbf{II}}$, Ar), 7.31 – 7.22 (m, 2H$^{\mathbf{I}}$+2H$^{\mathbf{II}}$, Ar), 7.18 – 7.14 (m, 2H$^{\mathbf{I}}$, Ar).

^{13}C{^{1}H} NMR (101 MHz, DMSO-d_6) δ 162.9, 151.1, 136.3, 132.5, 132.1, 131.8, 131.4, 130.9, 130.3, 129.5, 129.4, 129.3, 129.3, 129.2, 129.2, 129.1, 129.1, 129.0, 128.8, 128.6, 128.3, 127.0, 126.9, 126.7, 126.7, 122.6, 104.7.

HRMS (ESI) calcd for $C_{28}H_{18}Cl_2N_4O_3$ $[M+H]^+$ *m/z* 529.0829, found *m/z* 529.0829.

4-(4-Bromophenyl)-3-(4-chlorophenyl)-6,8-diphenyl-1-oxa-2,4,6,8-tetraazaspiro[4.4]non-2-ene-7,9-dione (**5c**)

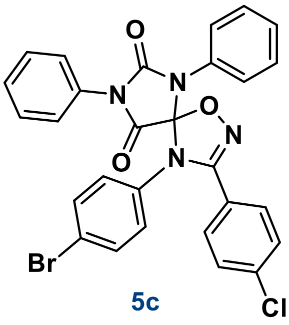

5c

Compound **5c** was obtained from dipolarophile **2c** (63 mg, 0.150 mmol), (*Z*)-4-chloro-*N*-hydroxybenzimidoyl chloride (**4a**, 31 mg, 0.165 mmol) as yellowish solid (*TDA*: 71 mg, 83% yield; *DMT*: 64 mg, 74% yield).

^{1}H NMR (400 MHz, $CDCl_3$) δ 7.56 – 7.52 (m, 2H), 7.50 – 7.42 (m, 4H), 7.42 – 7.34 (m, 4H), 7.33 – 7.29 (m, 2H), 7.27 – 7.23 (m, 2H), 7.18 – 7.15 (m, 2H), 6.74 – 6.70 (m, 2H).

^{13}C{^{1}H} NMR (101 MHz, $CDCl_3$) δ 164.3, 154.3, 151.6, 137.7, 133.7, 133.1, 132.9, 130.4, 129.6, 129.5, 129.4, 129.4, 129.0, 128.3, 128.3, 126.1, 125.8, 122.5, 121.5, 105.5.

HRMS (ESI) calcd for $C_{28}H_{18}BrClN_4O_3$ $[M+Na]^+$ *m/z* 595.0143, found *m/z* 595.0149.

4-(2-Bromophenyl)-3-(4-chlorophenyl)-6,8-diphenyl-1-oxa-2,4,6,8-tetraazaspiro[4.4]non-2-ene-7,9-dione (**5d**)

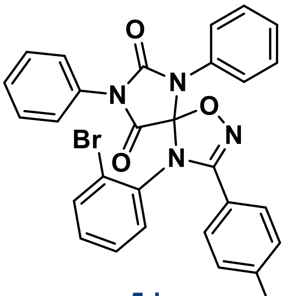

5d

Compound **5d** was obtained from dipolarophile **2d** (63 mg, 0.150 mmol), (*Z*)-4-chloro-*N*-hydroxybenzimidoyl chloride (**4a**, 31 mg, 0.165 mmol) as yellowish solid (*TDA*: 74 mg, 86% yield; *DMT*: 58 mg, 67% yield).

Mixture of isomers I and II (5:1 in DMSO-d_6):

^{1}H NMR (400 MHz, DMSO-d_6) δ 7.74 – 7.67 (m, 2H^{I}, Ar), 7.66 – 7.59 (m, 2H^{I}+2H^{II}, Ar), 7.57 – 7.52 (m, 1H^{I}+2H^{II}, Ar), 7.51 – 7.41 (m, 7H^{I}+6H^{II}, Ar), 7.40 – 7.33 (m, 2H^{I}+4H^{II}, Ar), 7.33 – 7.24 (m, 2H^{I}+4H^{II}, Ar), 7.20 – 7.13 (m, 2H^{I}, Ar).

^{13}C{^{1}H} NMR (101 MHz, DMSO-d_6) δ 162.9, 153.5, 151.1, 136.2, 134.7, 133.2, 132.5, 131.1, 130.4, 129.6, 129.5, 129.3, 129.2, 129.2, 129.0, 129.0, 128.6, 128.6, 127.3, 127.1, 126.7, 122.9, 122.9, 104.6.

HRMS (ESI) calcd for $C_{28}H_{18}BrClN_4O_3$ $[M+Na]^+$ *m*/*z* 595.0143, found *m*/*z* 595.0148.

Ethyl 4-(2-bromophenyl)-7,9-dioxo-6,8-diphenyl-1-oxa-2,4,6,8-tetraazaspiro[4.4]non-2-ene-3-carboxylate (**5e**)

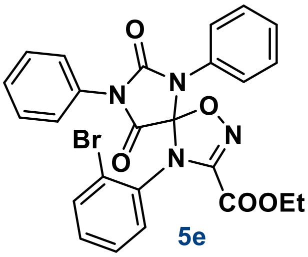

5e

Synthesis of compound **5e** was performed according to the general procedure from dipolarophile **2d** (63 mg, 0.150 mmol) and ethyl (*Z*)-2-chloro-2-(hydroxyimino)acetate (**4d**, 25 mg, 0.165 mmol). In both cases the target compound was not found in the reaction mixtures even after elongation of reaction time up to 3 weeks. The main product obtained via *DMT* was *N*-(2-bromophenyl)formamide **6** (24 mg, 80% yield)

*N-(2-bromophenyl)formamide (**6**) [9]*

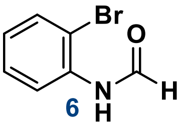

6

R_f 0.17 ($CHCl_3$).

^{1}H NMR (400 MHz, DMSO-d_6) δ 9.72 (s, 1H, <u>H</u>C=O), 8.35 (d, *J* = 1.8 Hz, 1H, NH), 8.01 (dd, *J* = 8.1, 1.6 Hz, 1H, Ar), 7.65 (dd, *J* = 8.1, 1.5 Hz, 1H, Ar), 7.39 – 7.33 (m, 1H, Ar), 7.09 (td, *J* = 7.7, 1.7 Hz, 1H, Ar).

^{13}C{^{1}H} NMR (101 MHz, DMSO-d_6) δ 160.4, 135.5, 132.7, 128.1, 126.2, 124.0, 114.5.

3-(4-Chlorophenyl)-6,8-diphenyl-4-(*p*-tolyl)-1-oxa-2,4,6,8-tetraazaspiro[4.4]non-2-ene-7,9-dione (**5f**)

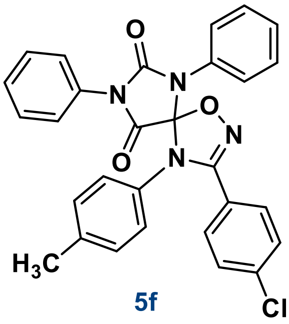

5f

Compound **5f** was obtained from dipolarophile **2e** (53 mg, 0.150 mmol), (*Z*)-4-chloro-*N*-hydroxybenzimidoyl chloride (**4a**, 31 mg, 0.165 mmol) as white solid (*TDA*: 65 mg, 85% yield; *DMT*: 60 mg, 79% yield).

^{1}H NMR (400 MHz, $CDCl_3$) δ 7.59 – 7.54 (m, 2H, Ar), 7.48 – 7.31 (m, 6H, Ar), 7.30 – 7.26 (m, 2H, Ar), 7.24 – 7.16 (m, 4H, Ar), 7.06 – 7.02 (m, 2H, Ar), 6.77 – 6.72 (m, 2H, Ar), 2.31 (s, 3H, CH_3).

^{13}C{^{1}H} NMR (101 MHz, $CDCl_3$) δ 164.59, 154.72, 151.76, 138.94, 137.33, 133.18, 131.73, 130.61, 130.45, 129.59, 129.43, 129.33, 129.12, 128.89, 128.04, 127.00, 126.22, 125.81, 121.91, 105.87, 21.21.

HRMS (ESI) calcd for $C_{29}H_{21}ClN_4O_3$ $[M+Na]^+$ *m*/*z* 531.1194, found *m*/*z* 531.1195.

3-(4-Chlorophenyl)-4-(4-methoxyphenyl)-6,8-diphenyl-1-oxa-2,4,6,8-tetraazaspiro[4.4]non-2-ene-7,9-dione (**5g**)

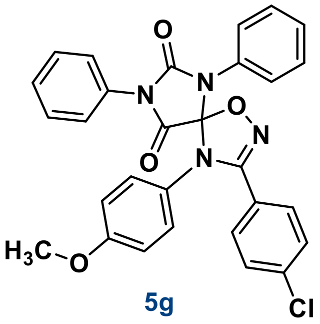

5g

Compound **5g** was obtained from dipolarophile **2f** (56 mg, 0.150 mmol), (*Z*)-4-chloro-*N*-hydroxybenzimidoyl chloride (**4a**, 31 mg, 0.165 mmol) as light yellow solid (*TDA*: 41 mg, 52% yield; *DMT*: 27 mg, 34% yield).

^{1}H NMR (400 MHz, $CDCl_3$) δ 7.62 – 7.57 (m, 2H, Ar), 7.47 – 7.41 (m, 4H, Ar), 7.41 – 7.37 (m, 1H, Ar), 7.37 – 7.32 (m, 1H, Ar), 7.30 – 7.27 (m, 2H, Ar), 7.24 – 7.16 (m, 4H, Ar), 6.83 – 6.78 (m, 2H, Ar), 6.77 – 6.72 (m, 2H, Ar), 3.77 (s, 3H, CH_3).

^{13}C{^{1}H} NMR (101 MHz, $CDCl_3$) δ 164.7, 159.7, 155.0, 151.7, 137.3, 133.3, 130.6, 129.6, 129.4, 129.3, 129.3, 129.1, 128.9, 127.9, 126.5, 126.2, 125.5, 121.8, 114.9, 106.0, 55.6.

HRMS (ESI) calcd for $C_{29}H_{21}ClN_4O_4$ $[M+Na]^+$ *m*/*z* 547.1146, found *m*/*z* 547.1144.

3-(4-Methoxyphenyl)-6,8-diphenyl-4-(*p*-tolyl)-1-oxa-2,4,6,8-tetraazaspiro[4.4]non-2-ene-7,9-dione (**5h**)

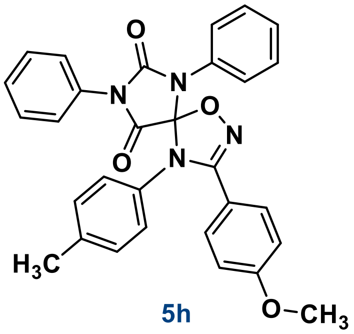

5h

Compound **5h** was obtained from dipolarophile **2e** (53 mg, 0.150 mmol), (*Z*)-*N*-hydroxy-4-methoxybenzimidoyl chloride (**4b**, 31 mg, 0.165 mmol) as yellowish solid (*TDA*: 55 mg, 72% yield; *DMT*: 35 mg, 46% yield).

^{1}H NMR (400 MHz, $CDCl_3$) δ 7.61 – 7.57 (m, 2H, Ar), 7.47 – 7.36 (m, 5H, Ar), 7.34 – 7.26 (m, 3H, Ar), 7.20 – 7.16 (m, 2H, Ar), 7.03 (d, *J* = 8.1 Hz, 2H, Ar), 6.79 – 6.72 (m, 4H, Ar), 3.75 (s, 3H, OCH_3), 2.30 (s, 3H, CH_3).

^{13}C{^{1}H} NMR (101 MHz, $CDCl_3$) δ 164.8, 161.6, 155.3, 151.8, 138.5, 133.3, 132.2, 130.7, 130.3, 129.9, 129.4, 129.3, 128.8, 127.9, 127.0, 126.2, 125.7, 115.4, 114.2, 105.7, 55.4, 21.2.

HRMS (ESI) calcd for $C_{30}H_{24}N_4O_4$ $[M+Na]^+$ *m*/*z* 527.1690, found *m*/*z* 527.1689.

Ethyl 2-(4-(4-bromophenyl)-3-(4-chlorophenyl)-7,9-dioxo-8-phenyl-1-oxa-2,4,6,8-tetraazaspiro[4.4]non-2-en-6-yl)acetate (**5i**)

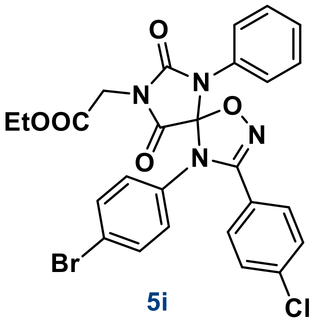

5i

Compound **5i** was obtained from dipolarophile **2h** (65 mg, 0.150 mmol), (*Z*)-4-chloro-*N*-hydroxybenzimidoyl chloride (**4a**, 31 mg, 0.165 mmol) as colorless oil (*TDA*: 80 mg, 91% yield).

R_f 0.38 (DCM).

^{1}H NMR (400 MHz, $CDCl_3$) δ 7.41 – 7.35 (m, 4H, Ar), 7.35 – 7.30 (m, 3H, Ar), 7.26 – 7.23 (m, 2H, Ar), 7.19 – 7.15 (m, 2H, Ar), 6.67 – 6.62 (m, 2H, Ar), 4.35 (s, 2H, NCH_2), 4.23 (qd, *J* = 7.2, 1.2 Hz, 2H, $O\underline{CH_2}CH_3$), 1.28 (t, *J* = 7.1 Hz, 3H, $OCH_2\underline{CH_3}$).

^{13}C{^{1}H} NMR (101 MHz, $CDCl_3$) δ 166.2, 165.1, 154.3, 151.8, 137.6, 133.4, 132.8, 129.6, 129.5, 129.3, 128.2, 128.0, 125.8, 121.9, 121.7, 105.9, 62.3, 40.0, 14.2.

HRMS (ESI) calcd for $C_{26}H_{20}BrClN_4O_5$ $[M+Na]^+$ *m*/*z* 605.0198, found *m*/*z* 605.0203.

Ethyl 2-(4-(4-bromophenyl)-3-(4-chlorophenyl)-7,9-dioxo-6-phenyl-1-oxa-2,4,6,8-tetraazaspiro[4.4]non-2-en-8-yl)acetate (**5j**)

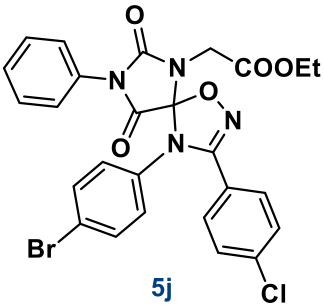

5j

Compound **5j** was obtained from dipolarophile **2i** (65 mg, 0.150 mmol), (*Z*)-4-chloro-*N*-hydroxybenzimidoyl chloride (**4a**, 31 mg, 0.165 mmol) as white solid (*TDA*: 22 mg, 25% yield).

R_f 0.31 (DCM).

^{1}H NMR (400 MHz, $CDCl_3$) δ 7.45 – 7.37 (m, 5H, Ar), 7.37 – 7.31 (m, 4H, Ar), 7.24 – 7.19 (m, 2H, Ar), 7.19 – 7.14 (m, 2H, Ar), 4.47 (d, *J* = 18.0 Hz, 1H, NCH_2), 4.29 – 4.18 (m, 2H, O$\underline{CH_2}$$CH_3$), 4.14 (d, *J* = 17.9 Hz, 1H, NCH_2), 1.29 – 1.25 (m, 3H, $OCH_2$$\underline{CH_3}$).

^{13}C{^{1}H} NMR (101 MHz, $CDCl_3$) δ 168.2, 163.7, 153.9, 152.8, 137.9, 134.9, 133.0, 130.4, 129.7, 129.5, 129.4, 129.0, 127.8, 125.9, 122.1, 121.7, 104.4, 62.2, 40.9, 14.2.

HRMS (ESI) calcd for $C_{26}H_{20}BrClN_4O_5$ $[M+Na]^+$ *m/z* 605.0198, found *m/z* 605.0202.

3-(4-Chlorophenyl)-4-(4-nitrophenyl)-6,8-diphenyl-1-oxa-2,4,6,8-tetraazaspiro[4.4]non-2-ene-7,9-dione (**5k**)

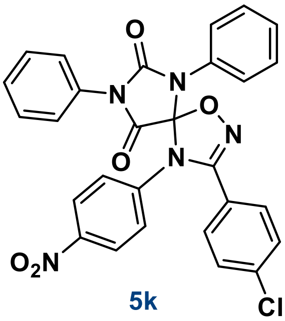

5k

Compound **5k** was obtained from dipolarophile **2g** (58 mg, 0.150 mmol), (*Z*)-4-chloro-*N*-hydroxybenzimidoyl chloride (**4a**, 31 mg, 0.165 mmol) as ivory solid (*TDA*: 37 mg, 46% yield; *DMT*: 42 mg, 51% yield).

^{1}H NMR (400 MHz, $CDCl_3$) δ 8.15 – 8.10 (m, 2H, Ar), 7.53 – 7.45 (m, 5H, Ar), 7.44 – 7.41 (m, 2H, Ar), 7.40 – 7.33 (m, 3H, Ar), 7.31 – 7.28 (m, 2H, Ar), 7.19 – 7.15 (m, 2H, Ar), 7.01 – 6.97 (m, 2H, Ar).

^{13}C{^{1}H} NMR (101 MHz, $CDCl_3$) δ 163.7, 153.5, 151.5, 146.3, 141.0, 138.2, 132.4, 130.3, 129.8, 129.7, 129.5, 129.4, 129.2, 128.8, 126.0, 126.0, 125.4, 125.3, 121.2, 105.2.

HRMS (ESI) calcd for $C_{28}H_{18}ClN_5O_5$ $[M+Na]^+$ *m/z* 562.0889, found *m/z* 562.0888.

3-(4-Nitrophenyl)-6,8-diphenyl-4-(*p*-tolyl)-1-oxa-2,4,6,8-tetraazaspiro[4.4]non-2-ene-7,9-dione (**5l**)

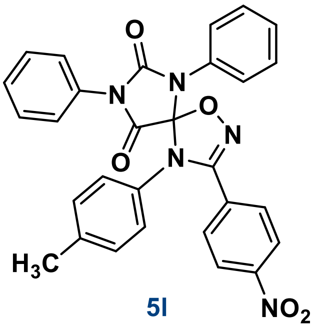

5l

Compound **5l** was obtained from dipolarophile **2e** (53 mg, 0.150 mmol), (*Z*)-*N*-hydroxy-4-nitrobenzimidoyl chloride (**4c**, 33 mg, 0.165 mmol) as beige solid (*TDA*: 74 mg, 93% yield; *DMT*: 70 mg, 88% yield).

^{1}H NMR (400 MHz, $CDCl_3$) δ 8.10 (d, *J* = 8.8 Hz, 2H, Ar), 7.56 (d, *J* = 7.9 Hz, 2H, Ar), 7.49 – 7.39 (m, 7H, Ar), 7.39 – 7.33 (m, 1H, Ar), 7.31 – 7.27 (m, 2H, Ar), 7.06 (d, *J* = 8.0 Hz, 2H, Ar), 6.75 (d, *J* = 8.3 Hz, 2H, Ar), 2.32 (s, 3H, CH_3).

^{13}C{^{1}H} NMR (101 MHz, $CDCl_3$) δ 164.4, 154.0, 151.7, 149.2, 139.5, 133.0, 131.3, 130.7, 130.5, 129.7, 129.5, 129.4, 129.3, 129.0, 128.3, 127.0, 126.2, 125.9, 123.9, 106.2, 21.3.

HRMS (ESI) calcd for $C_{29}H_{21}N_5O_5$ $[M+Na]^+$ *m/z* 542.1435, found *m/z* 542.1434.

X-ray crystallographic details

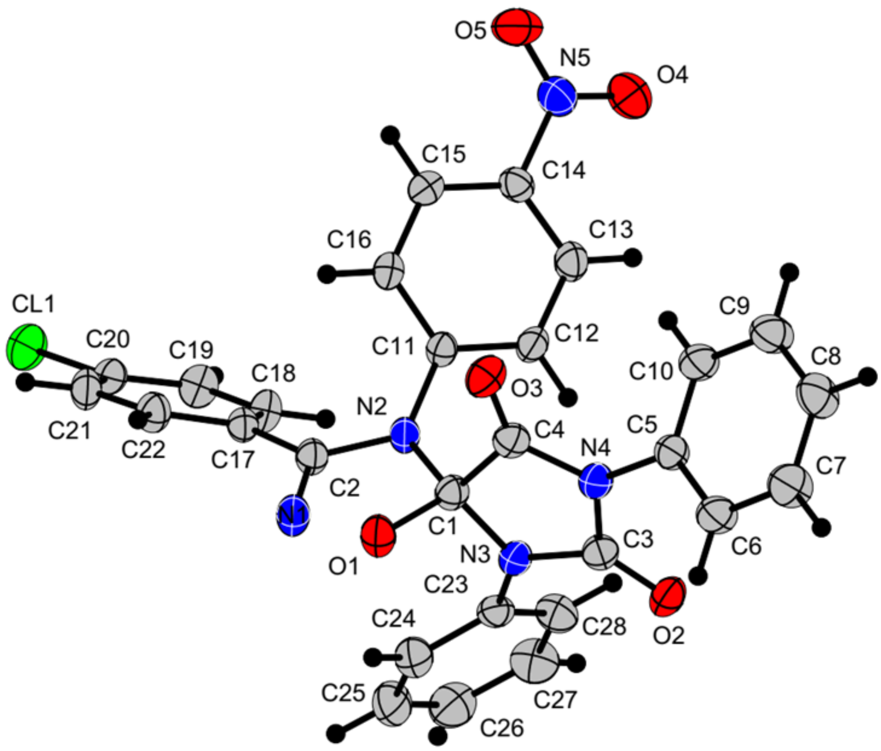

Figure S2. Thermal ellipsoid plot of the molecular structure of compound **5k** obtained by slow vaporization of a $CHCl_3$ solution at room temperature. Displacement ellipsoids are represented with 50% probability level.

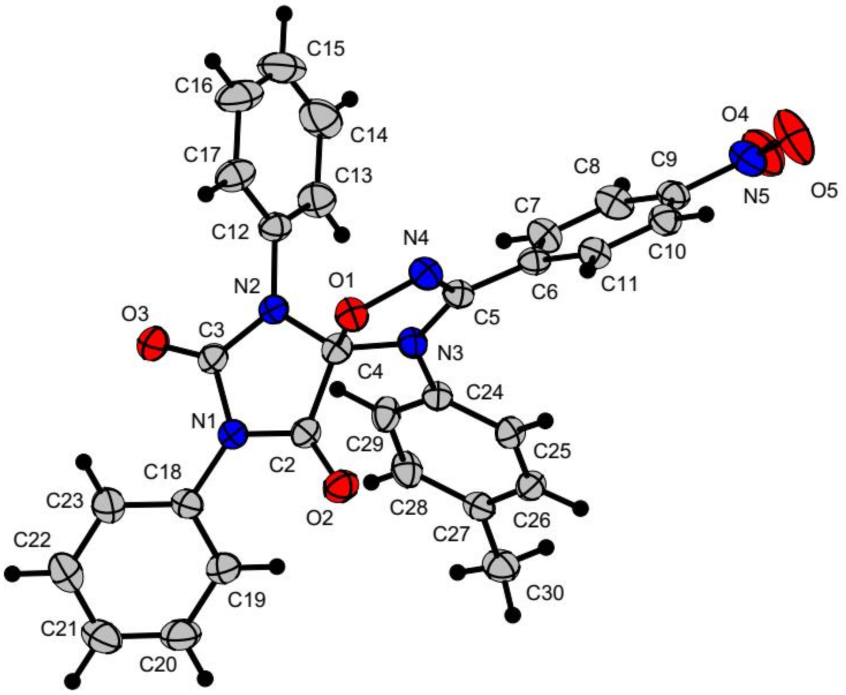

Figure S3. Thermal ellipsoid plot of the molecular structure of compound **5l** obtained by slow vaporization of a $CHCl_3$ solution at room temperature. Displacement ellipsoids are represented with 50% probability level.

Table S1. Crystal data and structure refinement for **5k** and **5l**.

Compound №	**5k**	**5l**
CCDC	2432465	2432466
Empirical formula	$C_{28}H_{18}ClN_5O_5$	$C_{29}H_{21}N_5O_5$
Formula weight	539.92	519.51
Wavelength	0.71073 Å	1.54186 Å
Crystal system	Monoclinic	Triclinic
Space group	*P* 21/*n*	*P* -1
Unit cell dimensions	a = 9.7584(5) Å<br>b = 16.6912(9) Å<br>c = 15.3640(8) Å	a = 10.4435(5) Å<br>b = 10.6436(5) Å<br>c = 12.0717(7) Å
	α = 90°<br>β = 93.861(4)°<br>γ = 90°	α = 92.685(7)°<br>β = 100.090(7)°<br>γ = 103.506(6)°
Volume	2496.8(2) $Å^3$	1279.15(12) $Å^3$
Z	4	2
Density (calculated)	1.436 Mg/m^3	1.349 Mg/m^3
Absorption coefficient	0.204 mm^{-1}	0.783 mm^{-1}
F(000)	1112	540
Theta range for data collection	1.804 to 27.147°	4.290 to 66.511°
Index ranges	-12<=h<=9<br>-21<=k<=21<br>-19<=l<=18	-12<=h<=12<br>-12<=k<=12<br>-13<=l<=3
Reflections collected	28940	9260
Independent reflections	5424 [R(int) = 0.0674]	4235 [R(int) = 0.0545]
Completeness to	theta = 25.242°<br>99.9 %	theta = 66.511°<br>93.5 %
Data / restraints / parameters	5424 / 0 / 353	4235 / 0 / 354
Goodness-of-fit on F2	0.817	0.903
Final R indices [I>2sigma(I)]	R1 = 0.0393<br>wR2 = 0.0650	R1 = 0.0555<br>wR2 = 0.1389
R indices (all data)	R1 = 0.1242<br>wR2 = 0.0823	R1 = 0.0741<br>wR2 = 0.1487
Extinction coefficient	0.0019(2)	0.0180(14)
Largest diff. peak and hole	0.183 and -0.176 e.$Å^{-3}$	0.228 and -0.265 e.$Å^{-3}$

Cytotoxicity evaluation

To evaluate the cytotoxicity of compounds in vitro, we acted in a manner analogous to [1] and placed cells in 4–7 × 10^3 cells/mL concentrations in 96-well culture plates for 24 h. Cells were counted after treatment with Trypan blue solution (0.4%). They were then exposed to different concentrations of compounds in two-fold serial (50–100 μM) dilutions in pre-incubated cells at 37 °C for 72 h. In control wells with untreated cells, only (DMSO + PBS) were added. Cell viability was measured by the standard MTT test [10] The absorbance was measured at 540 nm using a Multiskan™ FC microplate photometer and Skanlt software 6.1 RE for microplate reader, both from Thermo Scientific (Waltham, MA, USA).

In vitro experiments were carried out in triplicate. Graphpad prism version 9.0 was used to determine the IC_{50}. The data of IC_{50} are presented as mean ± standard deviation (SD).

Table S2. IC_{50} values obtained from the MTT test.

**Compound №**	**IC_{50} ± SD, μM**
5d	32,30±0,003 (R^2=0,99)
5f	≥50
5g	30,56±0,007 (R^2=1)
5h	39,68±0,05 (R^2=0,96)
5l	44,72±0,0007 (R^2=1)

References

(1) Kuznetsova, J. V.; Tkachenko, V. T.; Petrovskaya, L. M.; Filkina, M. E.; Shybanov, D. E.; Grishin, Y. K.; Roznyatovsky, V. A.; Tafeenko, V. A.; Pestretsova, A. S.; Yakovleva, V. A.; Pokrovsky, V. S.; Kukushkin, M. E.; Beloglazkina, E. K. *Int J Mol Sci* **2023**, *25*, 18. doi:10.3390/ijms25010018

(2) Watanabe, N.; Hamano, M.; Todaka, S.; Asaeda, T.; Ijuin, H. K.; Matsumoto, M. *Journal of Organic Chemistry* **2012**, *77*, 632–639. doi:10.1021/jo202304x

(3) Liu, K.-C.; Shelton, B. R.; Howe, R. K. *J Org Chem* **1980**, *45*, 3916–3918. doi:10.1021/jo01307a039

(4) Kozikowski, A. P.; Adamcz, M. *J Org Chem* **1983**, *48*, 366–372. doi:10.1021/jo00151a017

(5) Chen, Y.; Cantillo, D.; Kappe, C. O. *European J Org Chem* **2019**, *2019*, 2163–2171. doi:10.1002/ejoc.201900231

(6) Kitahara, K.; Toma, T.; Shimokawa, J.; Fukuyama, T. *Org Lett* **2008**, *10*, 2259–2261. doi:10.1021/ol800677p

(7) Castellano, S.; Kuck, D.; Viviano, M.; Yoo, J.; López-Vallejo, F.; Conti, P.; Tamborini, L.; Pinto, A.; Medina-Franco, J. L.; Sbardella, G. *J Med Chem* **2011**, *54*, 7663–7677. doi:10.1021/jm2010404

(8) Shybanov, D. E.; Filkina, M. E.; Kukushkin, M. E.; Grishin, Y. K.; Roznyatovsky, V. A.; Zyk, N. V.; Beloglazkina, E. K. *New Journal of Chemistry* **2022**, *46*, 18575–18586. doi:10.1039/d2nj03756d

(9) Ansari, Mohd. I.; Hussain, Mohd. K.; Yadav, N.; Gupta, P. K.; Hajela, K. *Tetrahedron Lett* **2012**, *53*, 2063–2065. doi:10.1016/j.tetlet.2012.02.027

(10) Bank, U.; Reinhold, D.; Ansorge, S. *Allerg Immunol (Leipz)* **1991**, *37*, 119–123

^{1}H and ^{13}C spectra for compounds 5a–l

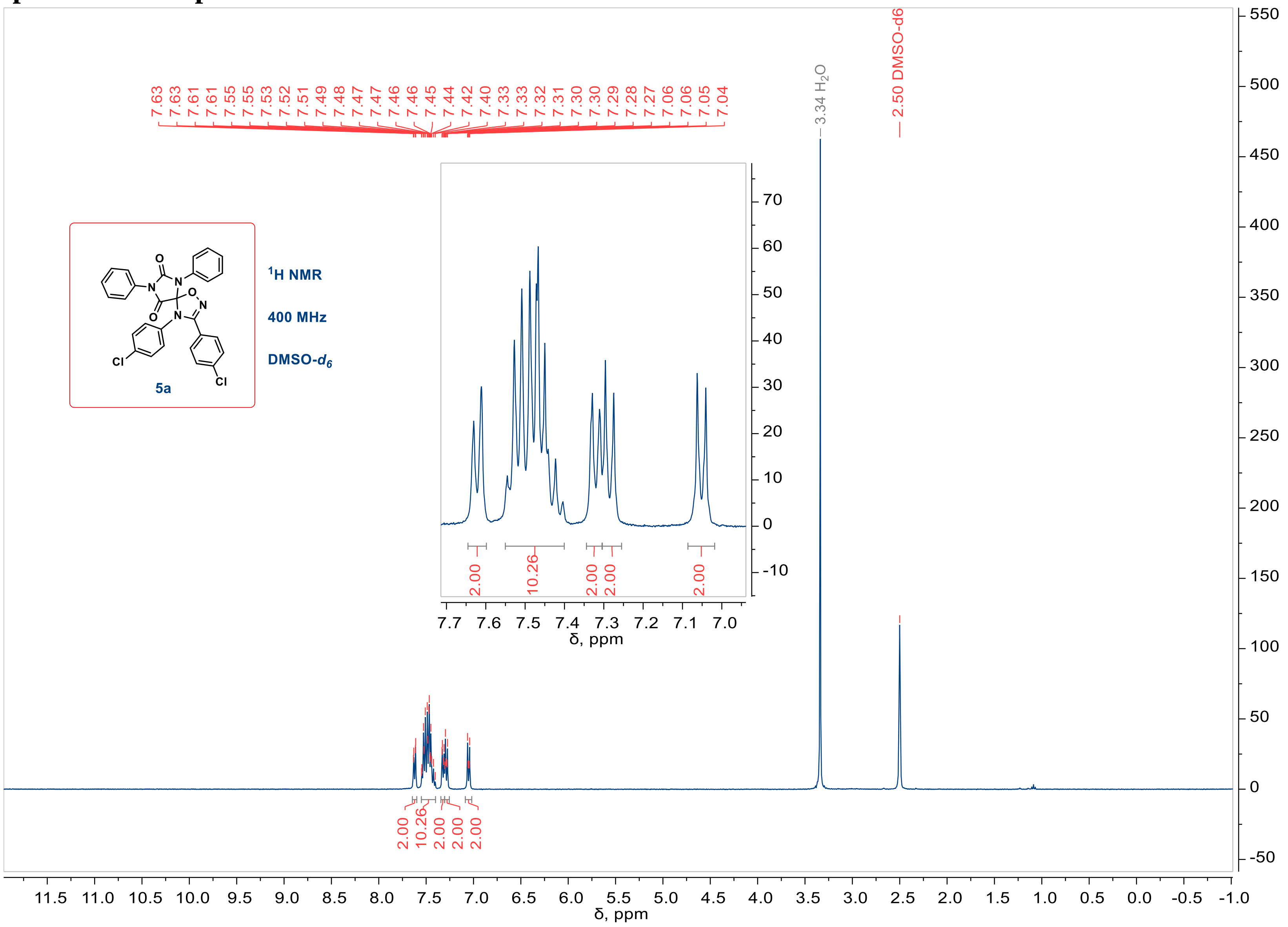

Figure S4. ^{1}H NMR spectrum of compound **5a** (DMSO-d_6, 400 MHz).

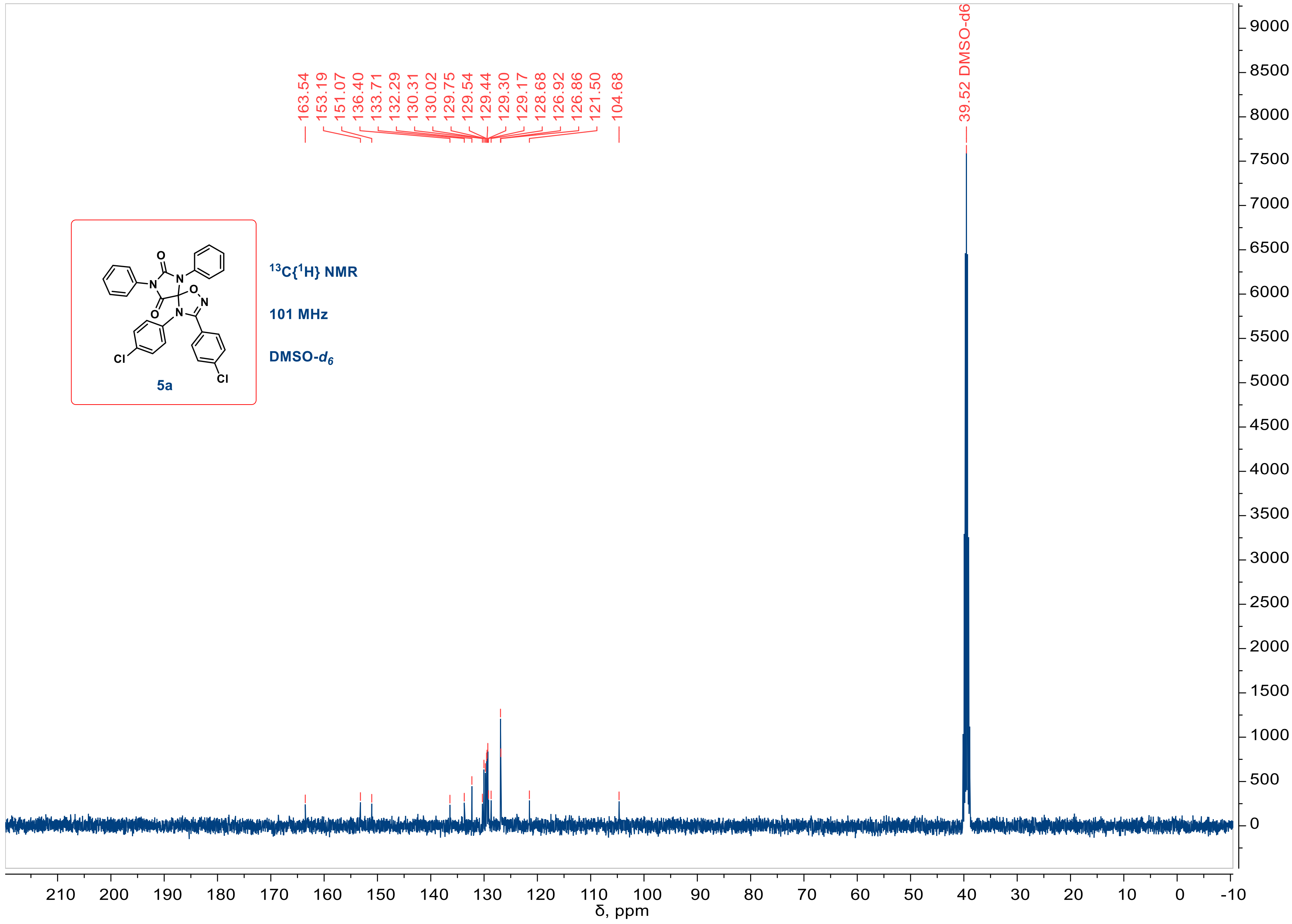

Figure S5. ^{13}C{1H} NMR spectrum of compound **5a** (DMSO-d_6, 101 MHz).

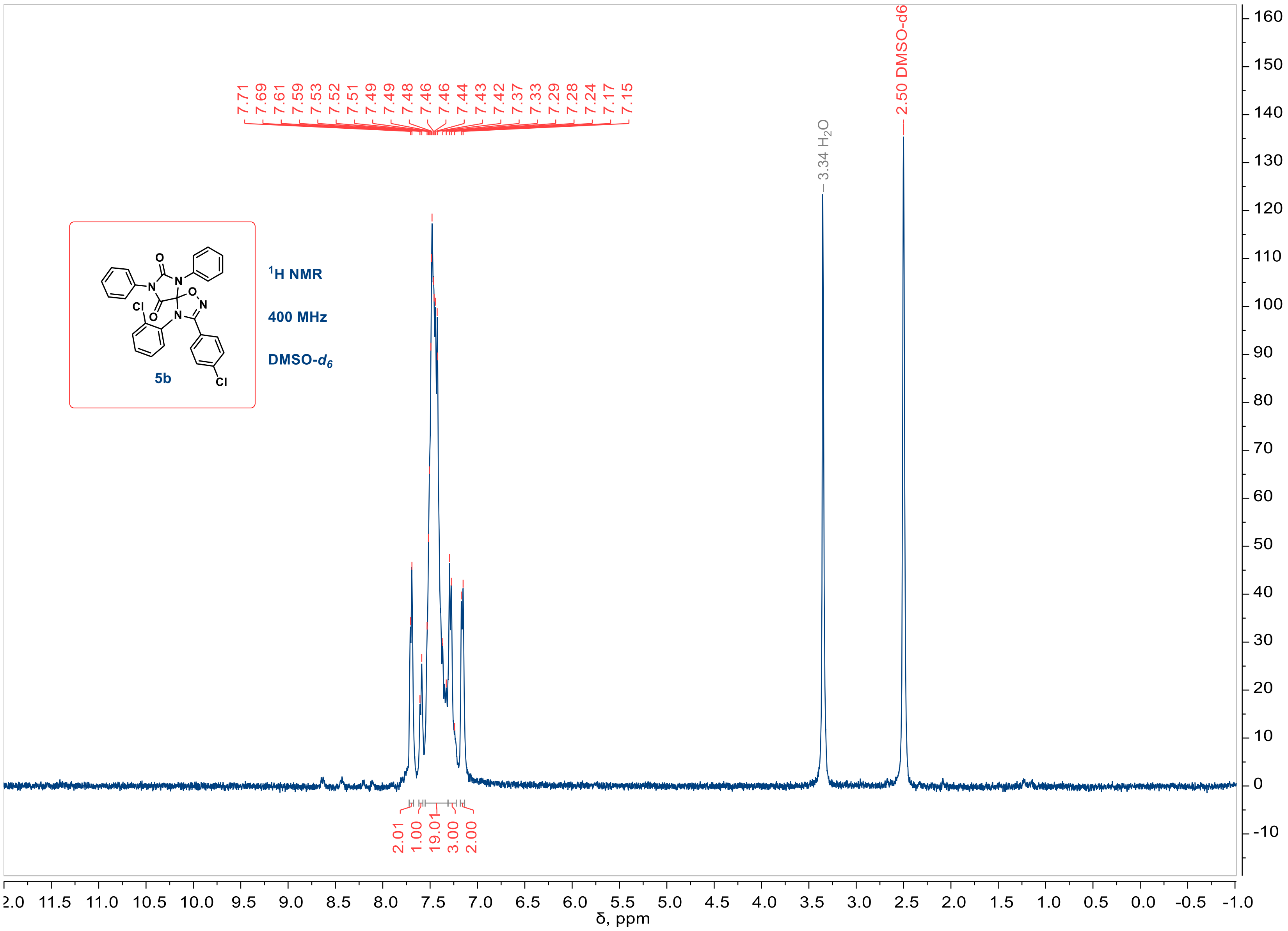

Figure S6. ^{1}H NMR spectrum of compound **5b** (DMSO-d_6, 400 MHz).

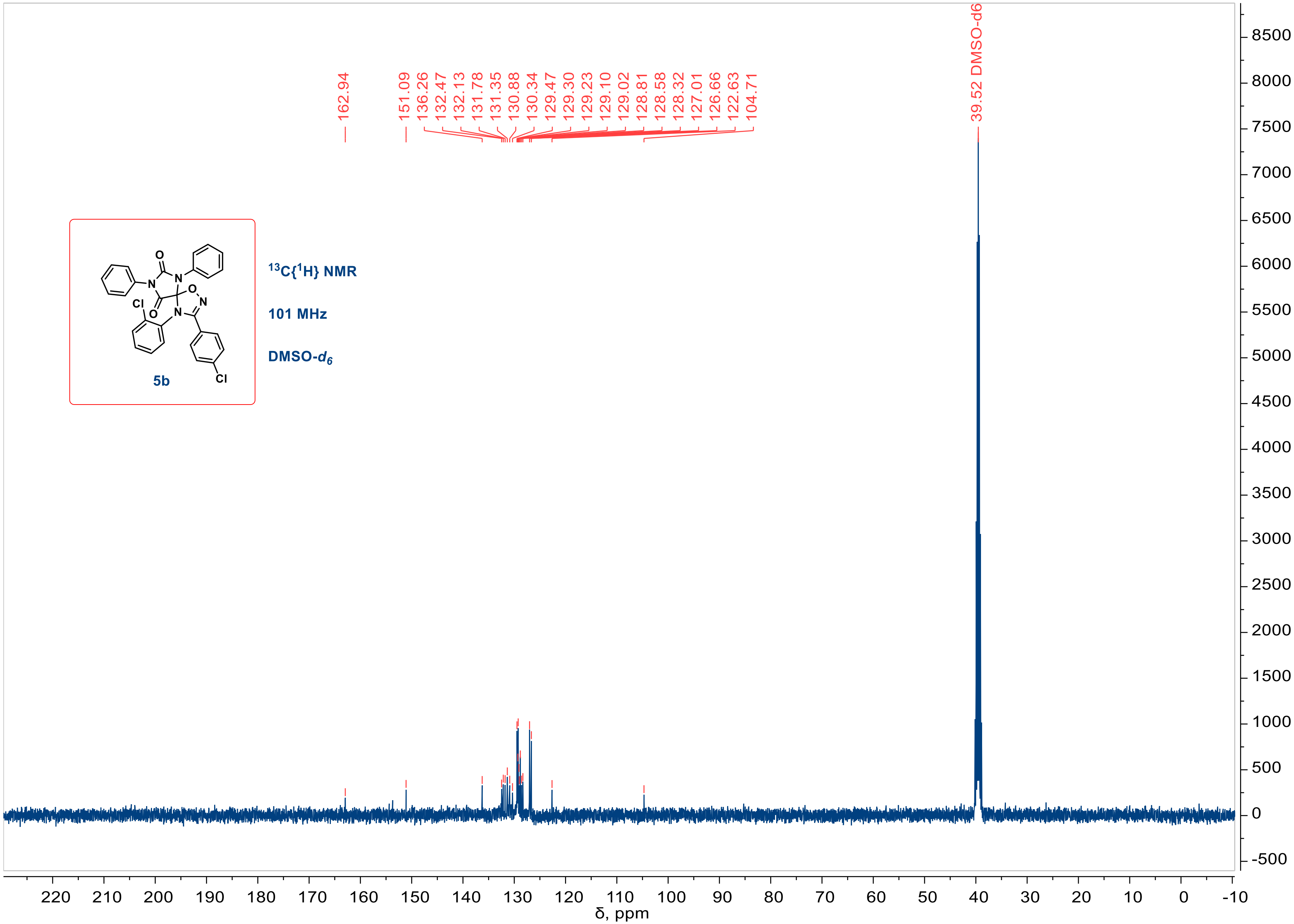

Figure S7. ^{13}C{1H} NMR spectrum of compound **5b** (DMSO-d_6, 101 MHz).

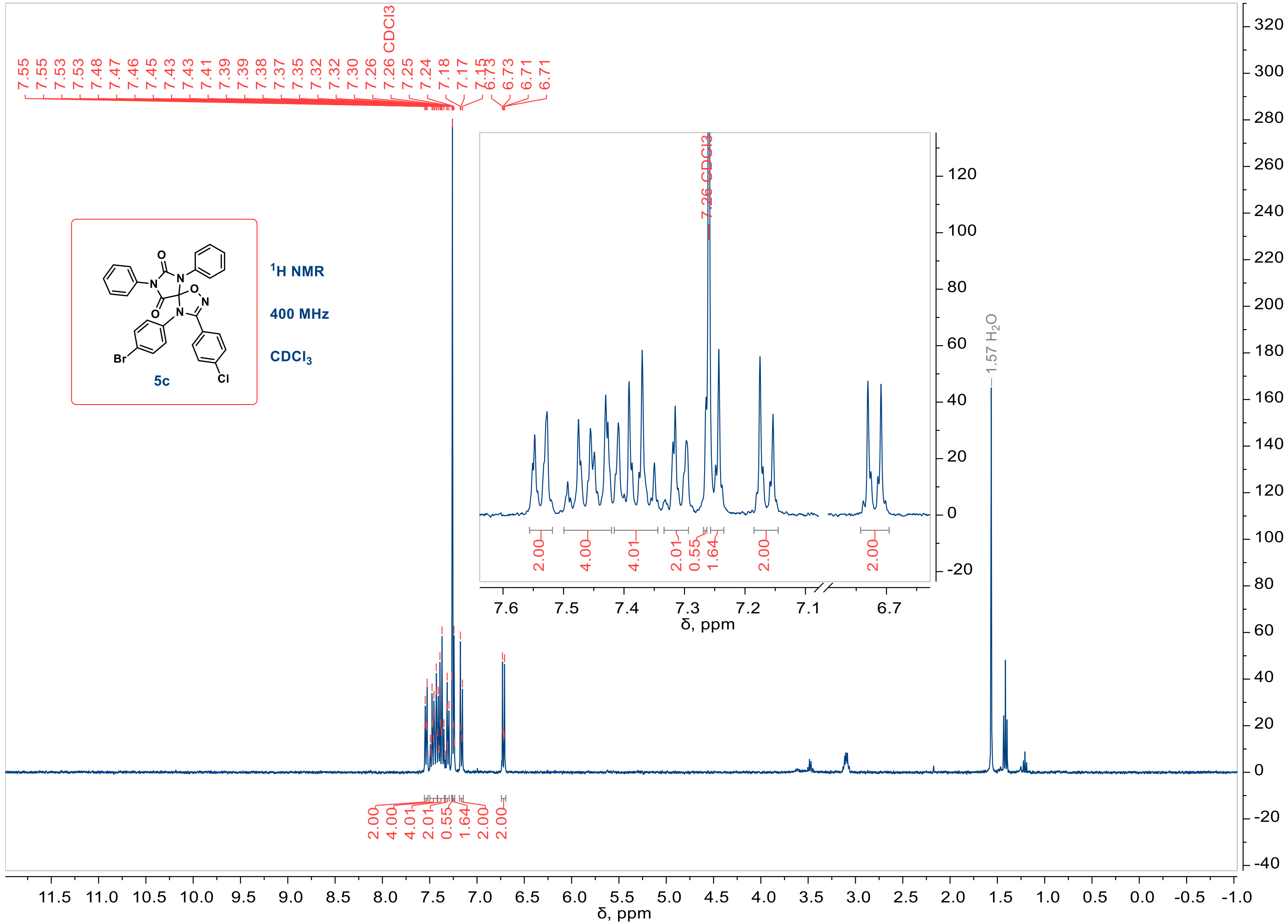

Figure S8. ^{1}H NMR spectrum of compound **5c** ($CDCl_3$, 400 MHz).

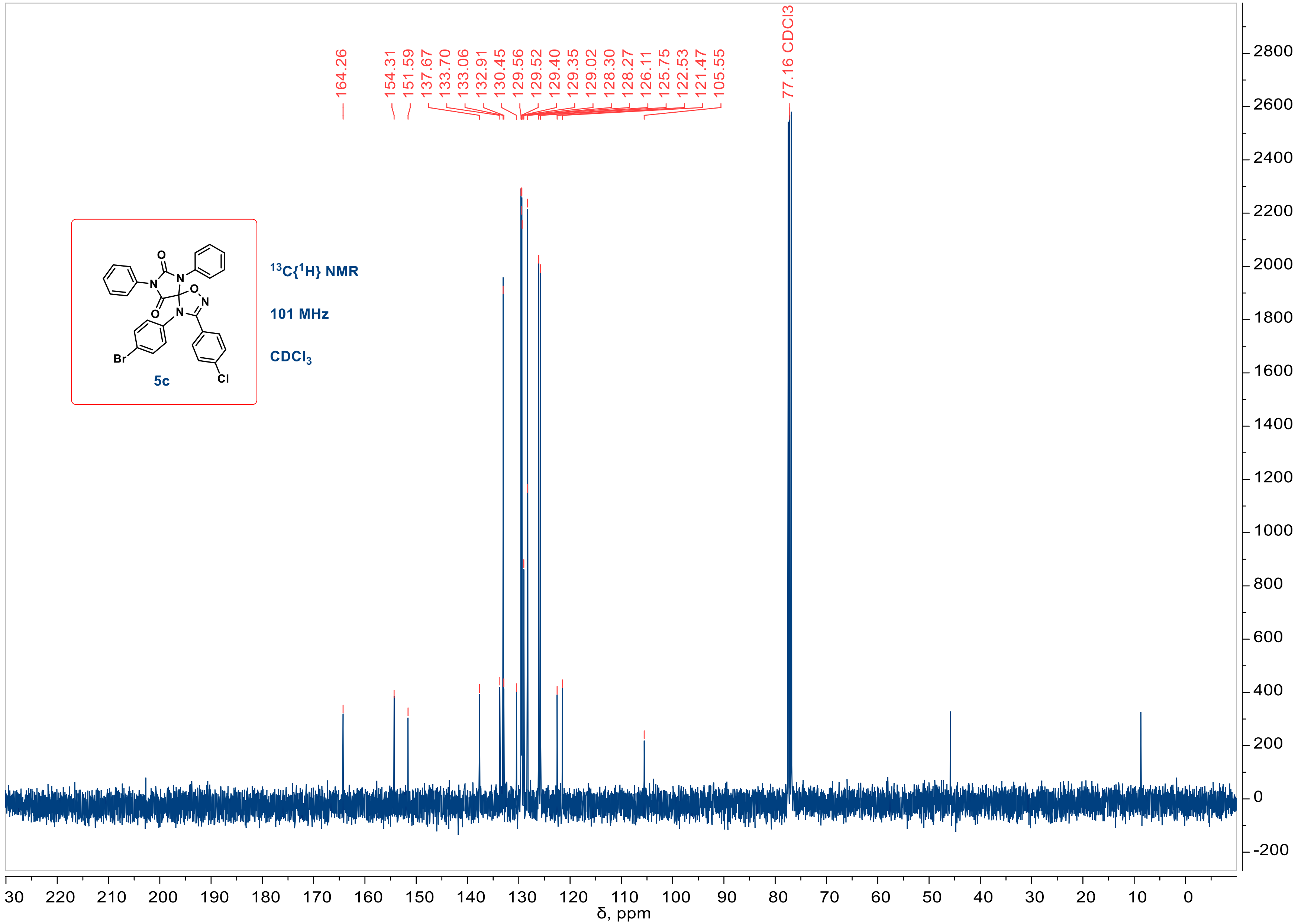

Figure S9. ^{13}C{1H} NMR spectrum of compound **5c** ($CDCl_3$, 101 MHz).

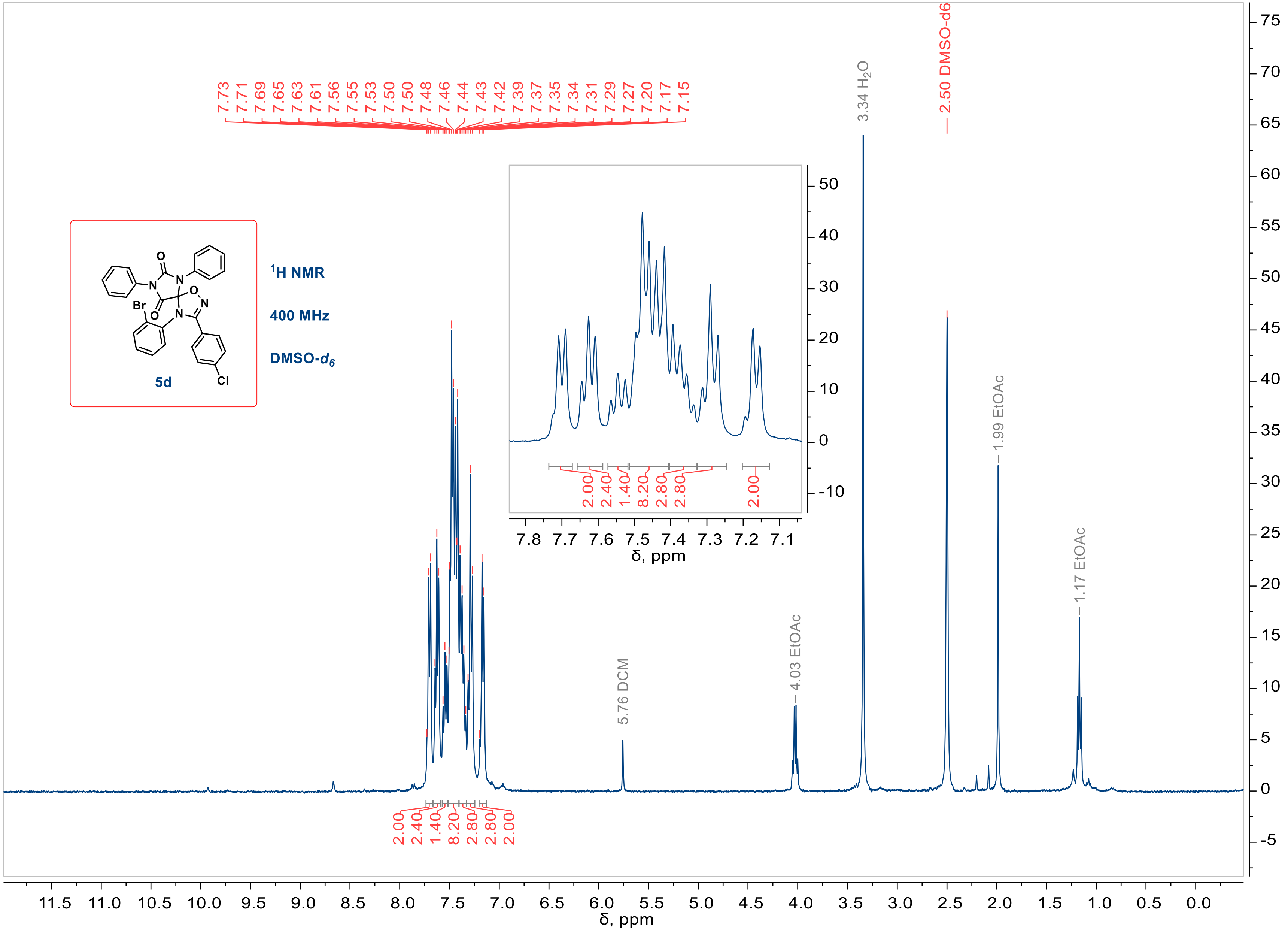

Figure S10. ¹H NMR spectrum of compound **5d** (DMSO-d_6, 400 MHz).

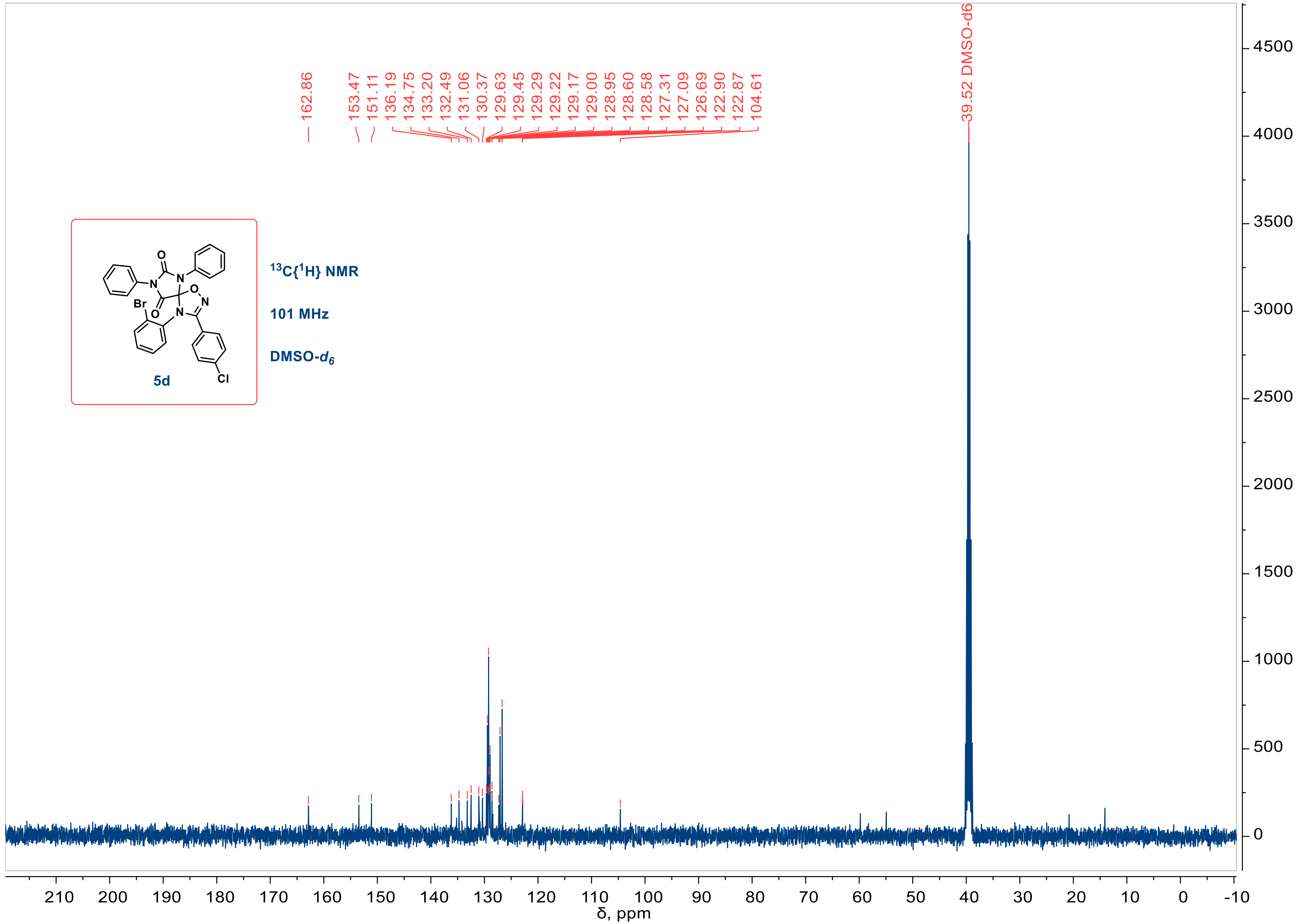

Figure S11. ^{13}C{1H} NMR spectrum of compound **5d** (DMSO-d_6, 101 MHz).

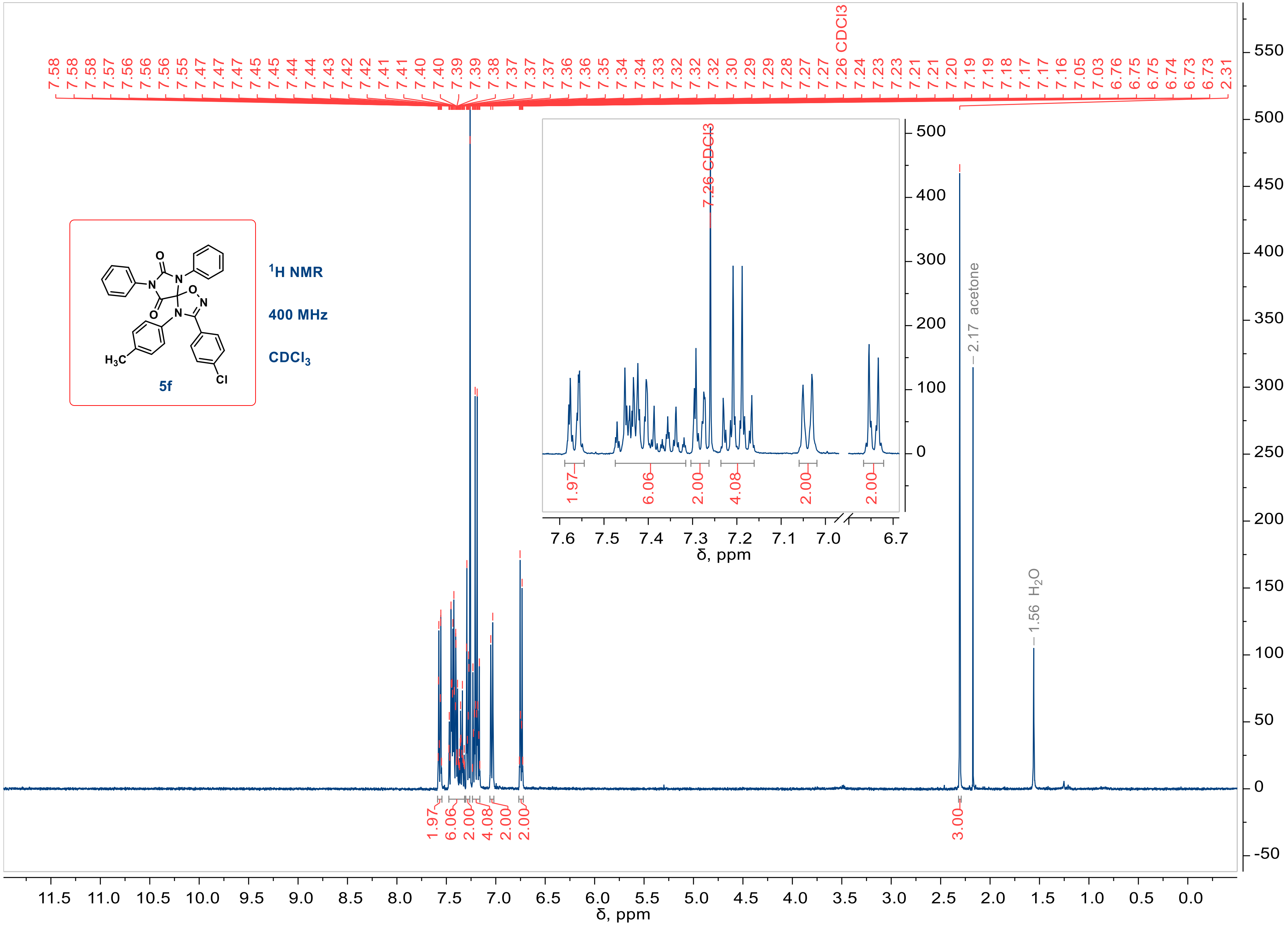

Figure S12. ^{1}H NMR spectrum of compound **5f** ($CDCl_3$, 400 MHz).

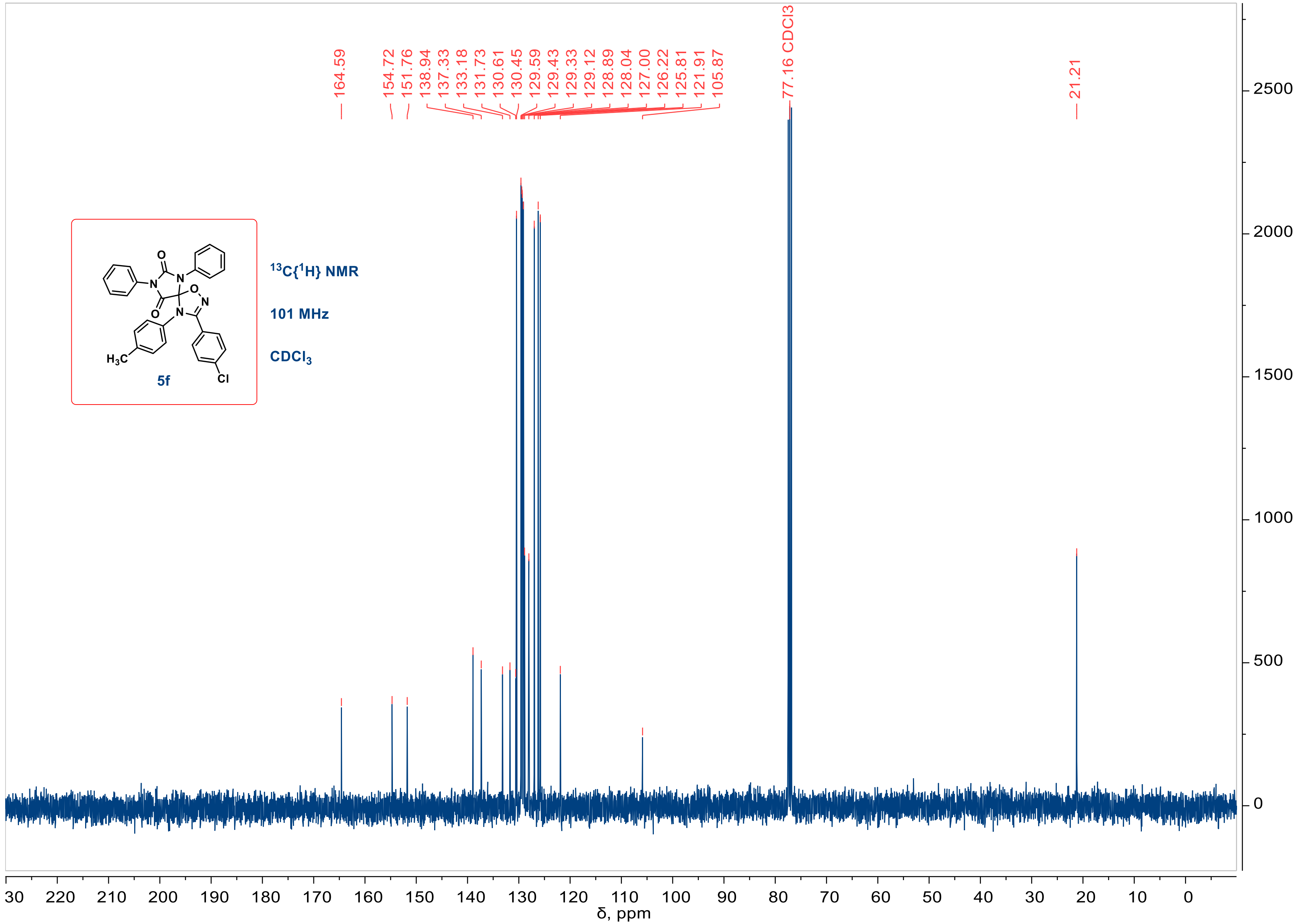

Figure S13. ^{13}C{1H} NMR spectrum of compound **5f** ($CDCl_3$, 101 MHz).

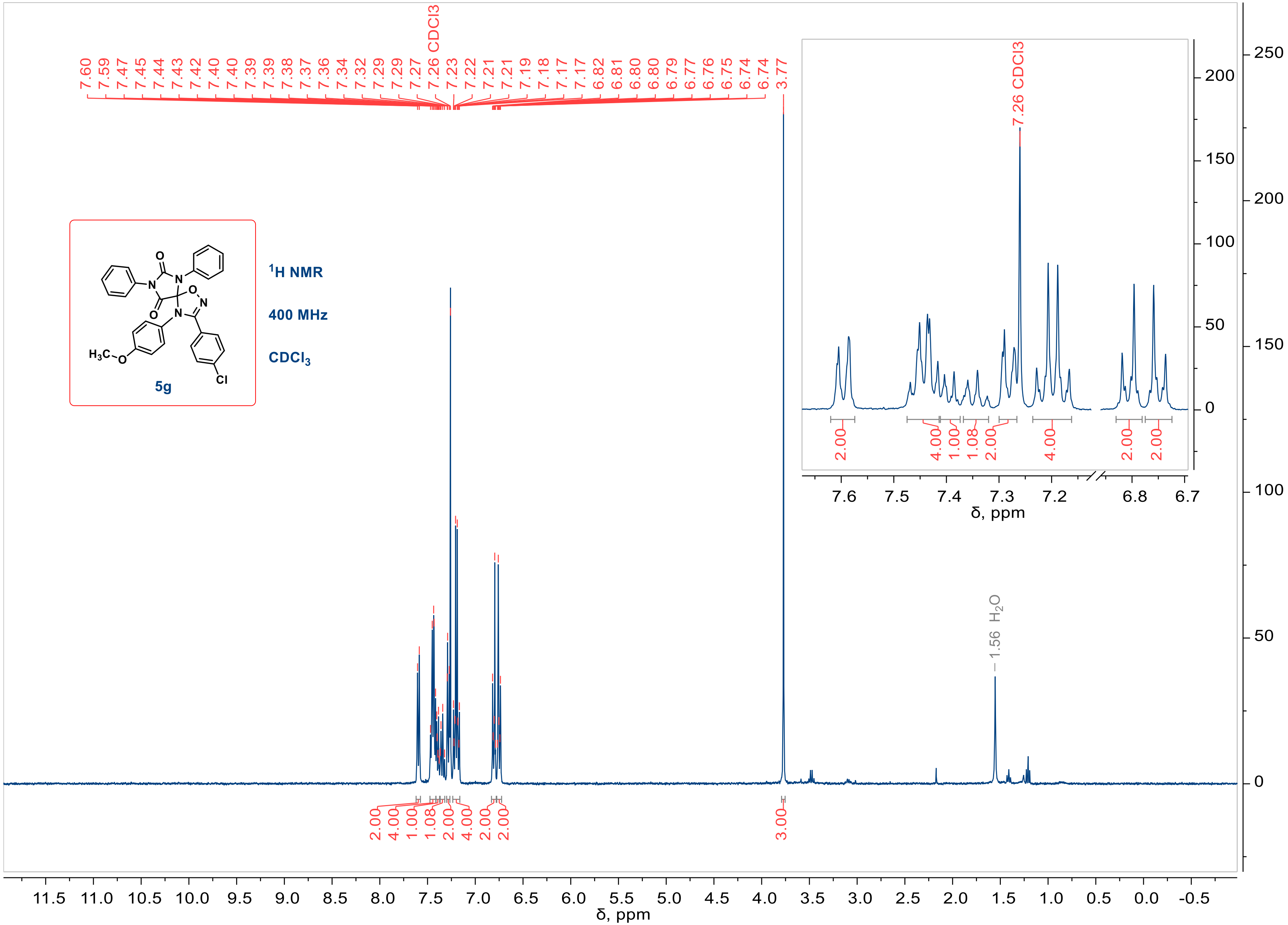

Figure S14. ¹H NMR spectrum of compound **5g** ($CDCl_3$, 400 MHz).

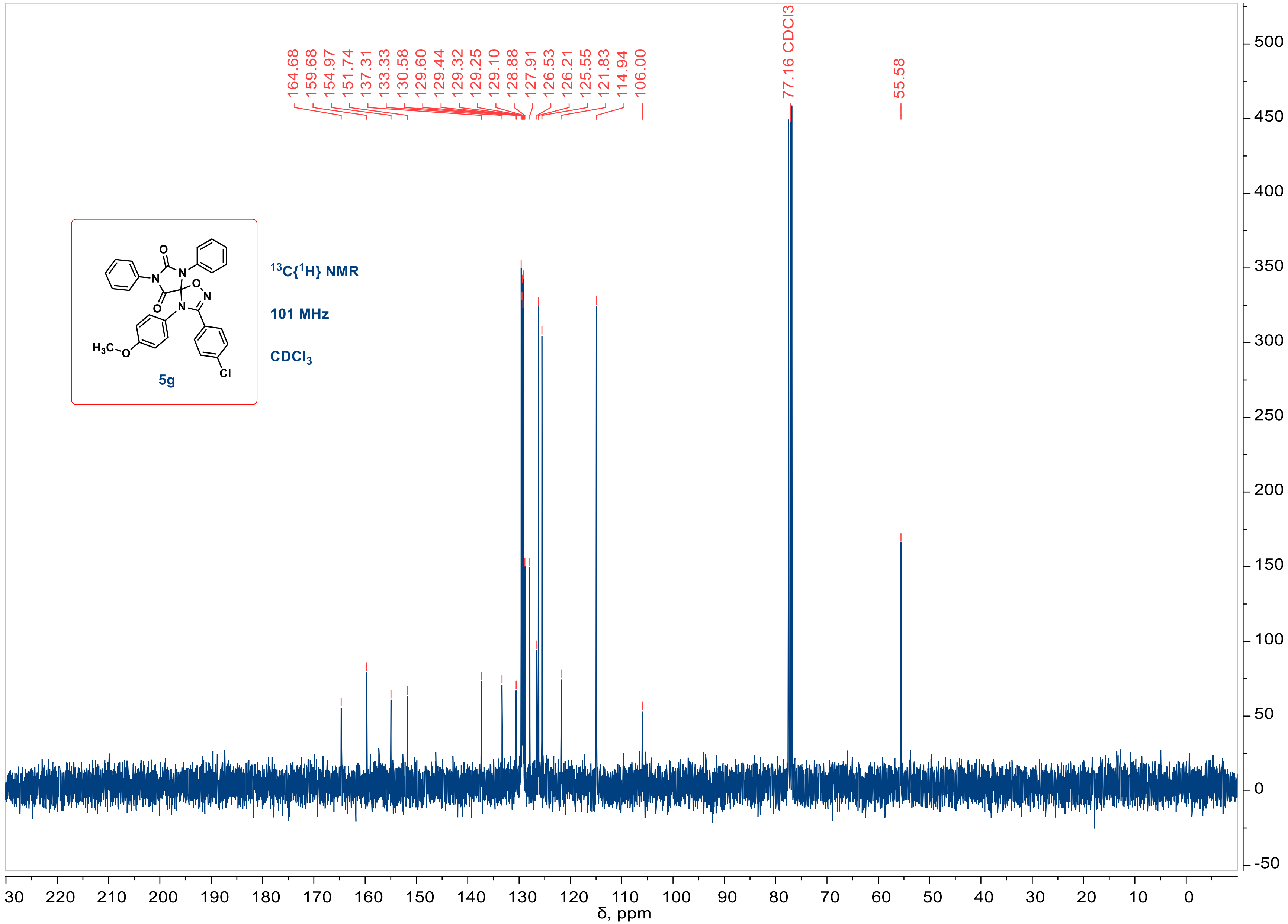

Figure S15. ^{13}C{1H} NMR spectrum of compound **5f** ($CDCl_3$, 101 MHz).

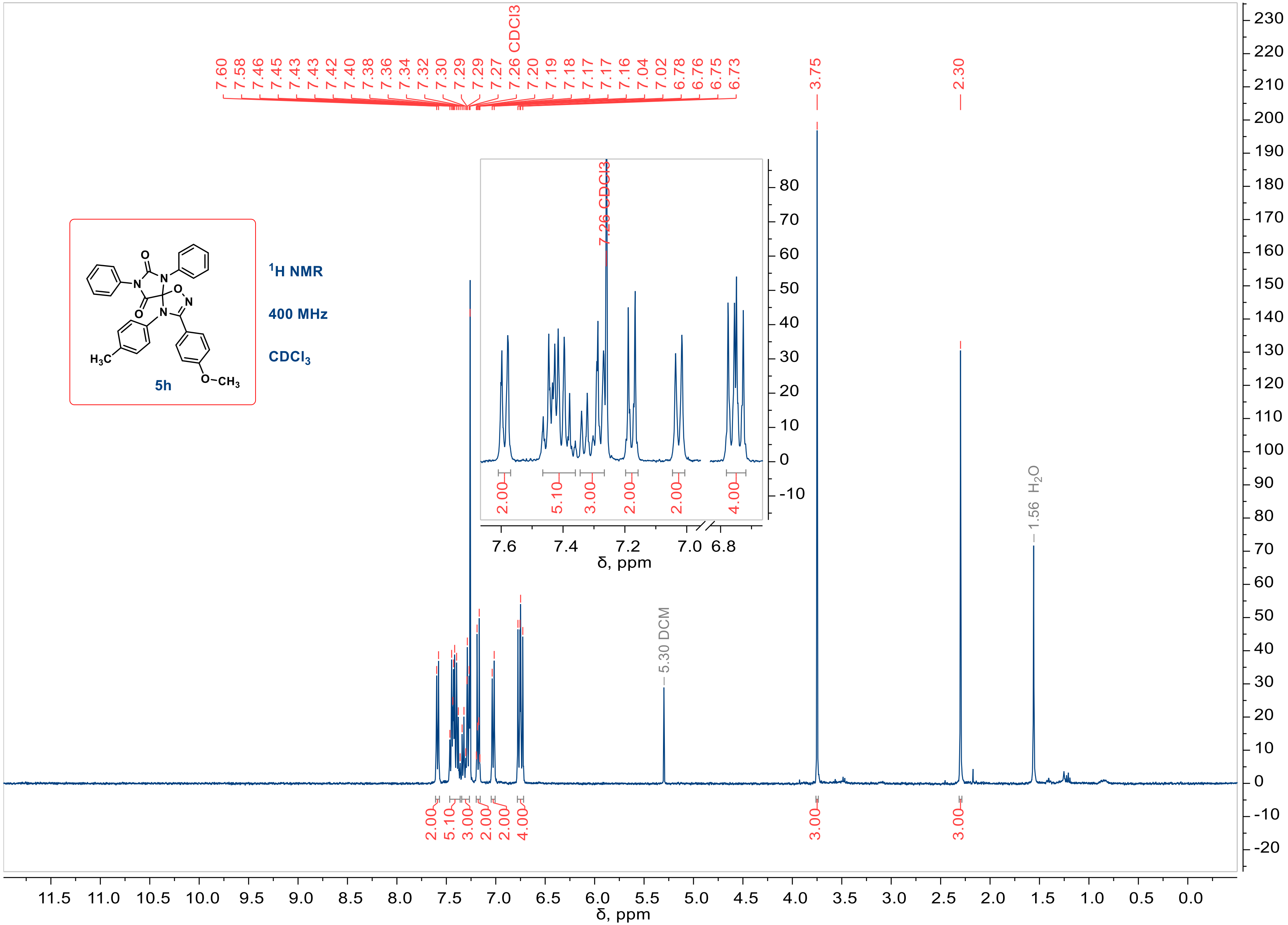

Figure S16. ^{1}H NMR spectrum of compound **5h** ($CDCl_3$, 400 MHz).

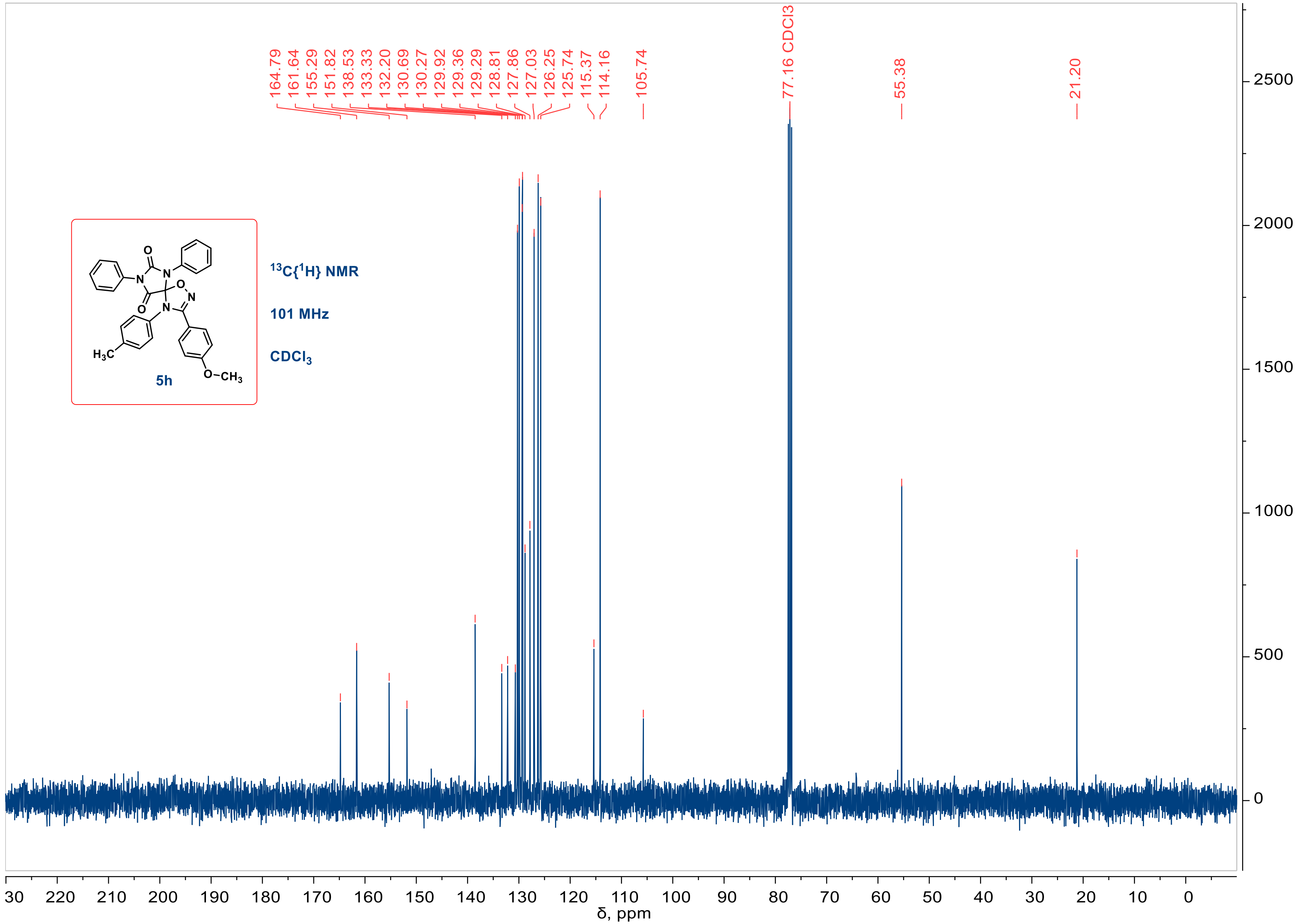

Figure S17. $^{13}C\{1H\}$ NMR spectrum of compound **5h** ($CDCl_3$, 101 MHz).

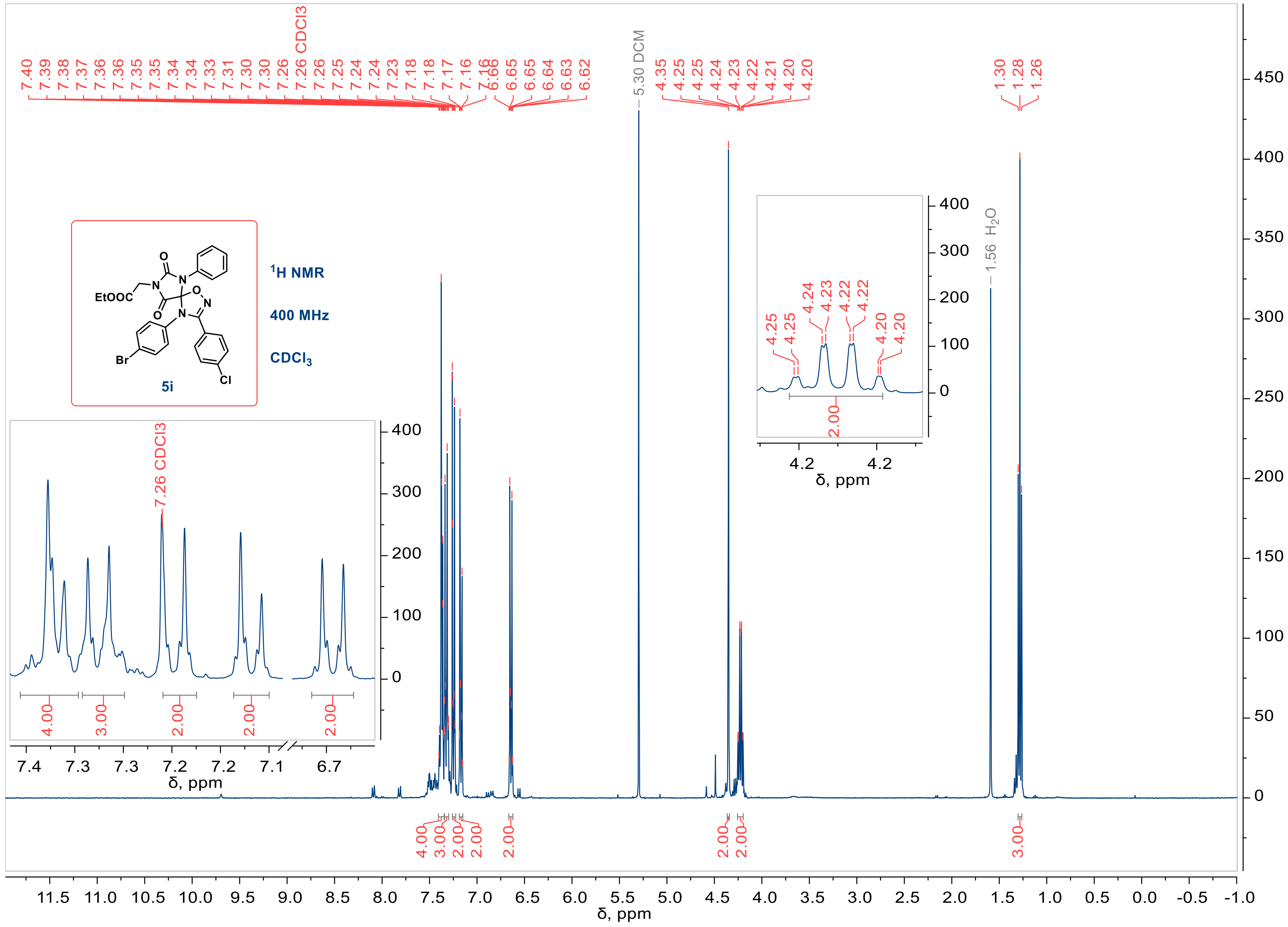

Figure S18. ^{1}H NMR spectrum of compound **5i** ($CDCl_3$, 400 MHz).

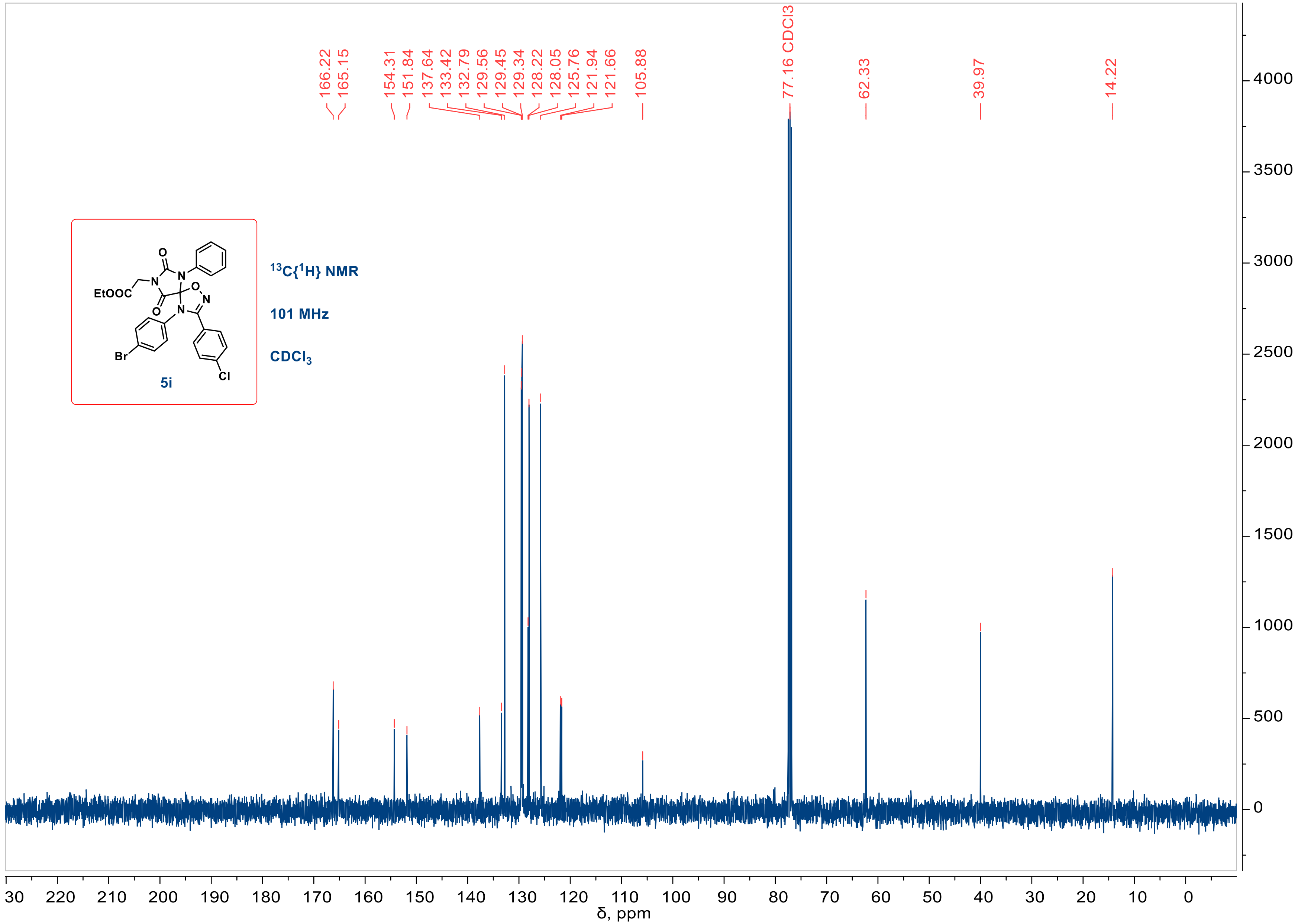

Figure S19. ^{13}C{1H} NMR spectrum of compound **5i** ($CDCl_3$, 101 MHz).

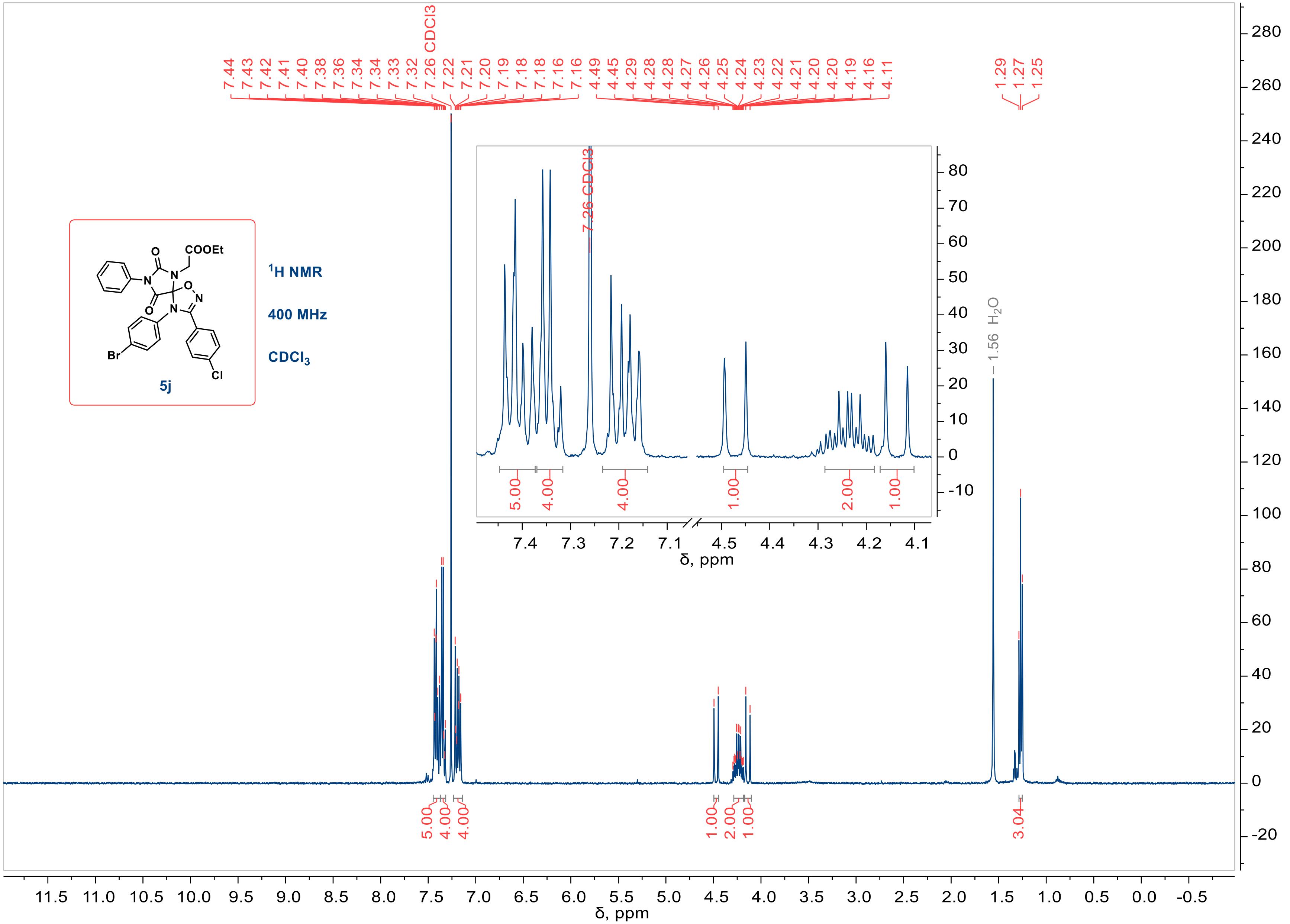

Figure S20. ^{1}H NMR spectrum of compound **5j** ($CDCl_3$, 400 MHz).

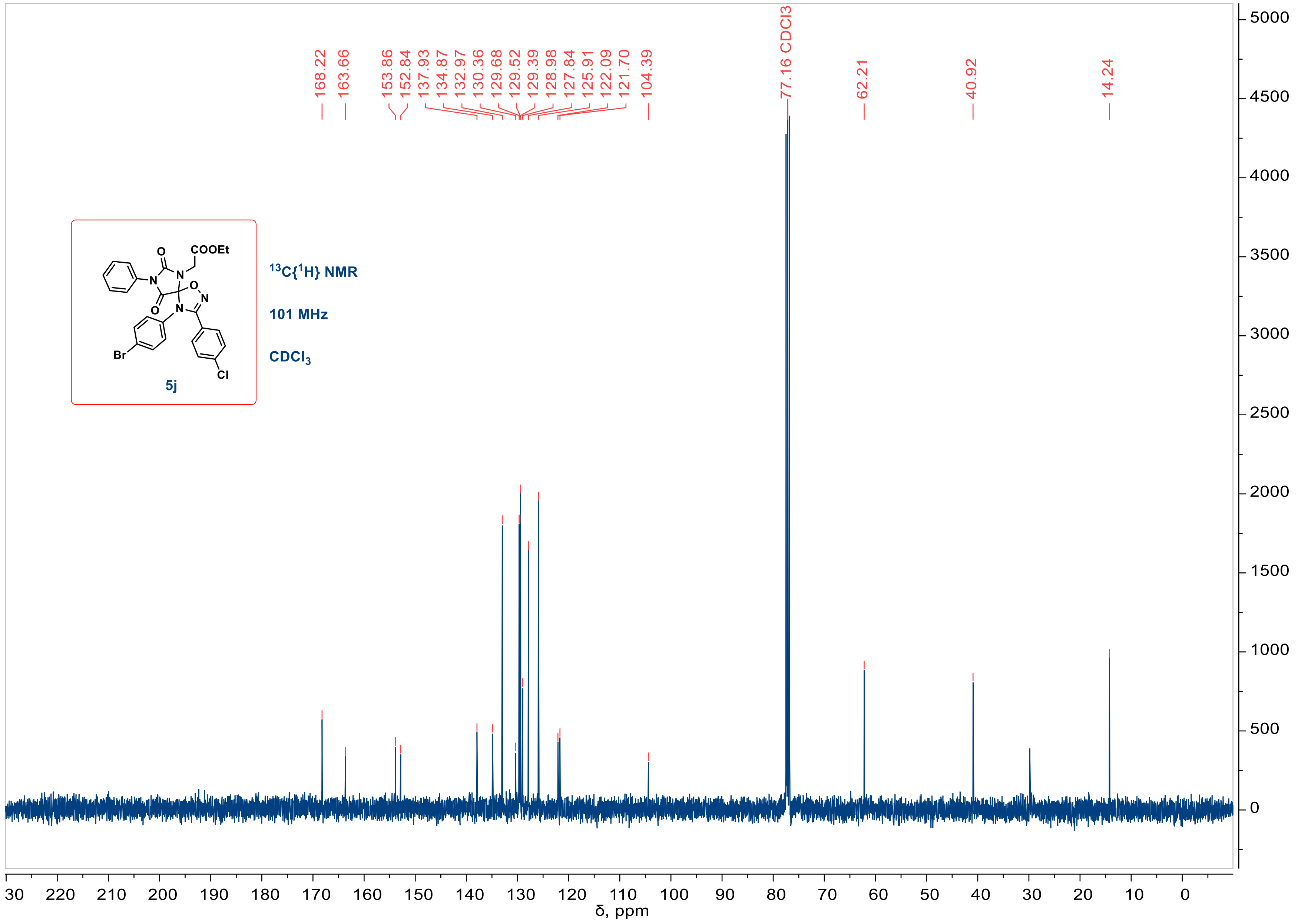

Figure S21. ^{13}C{1H} NMR spectrum of compound **5j** ($CDCl_3$, 101 MHz).

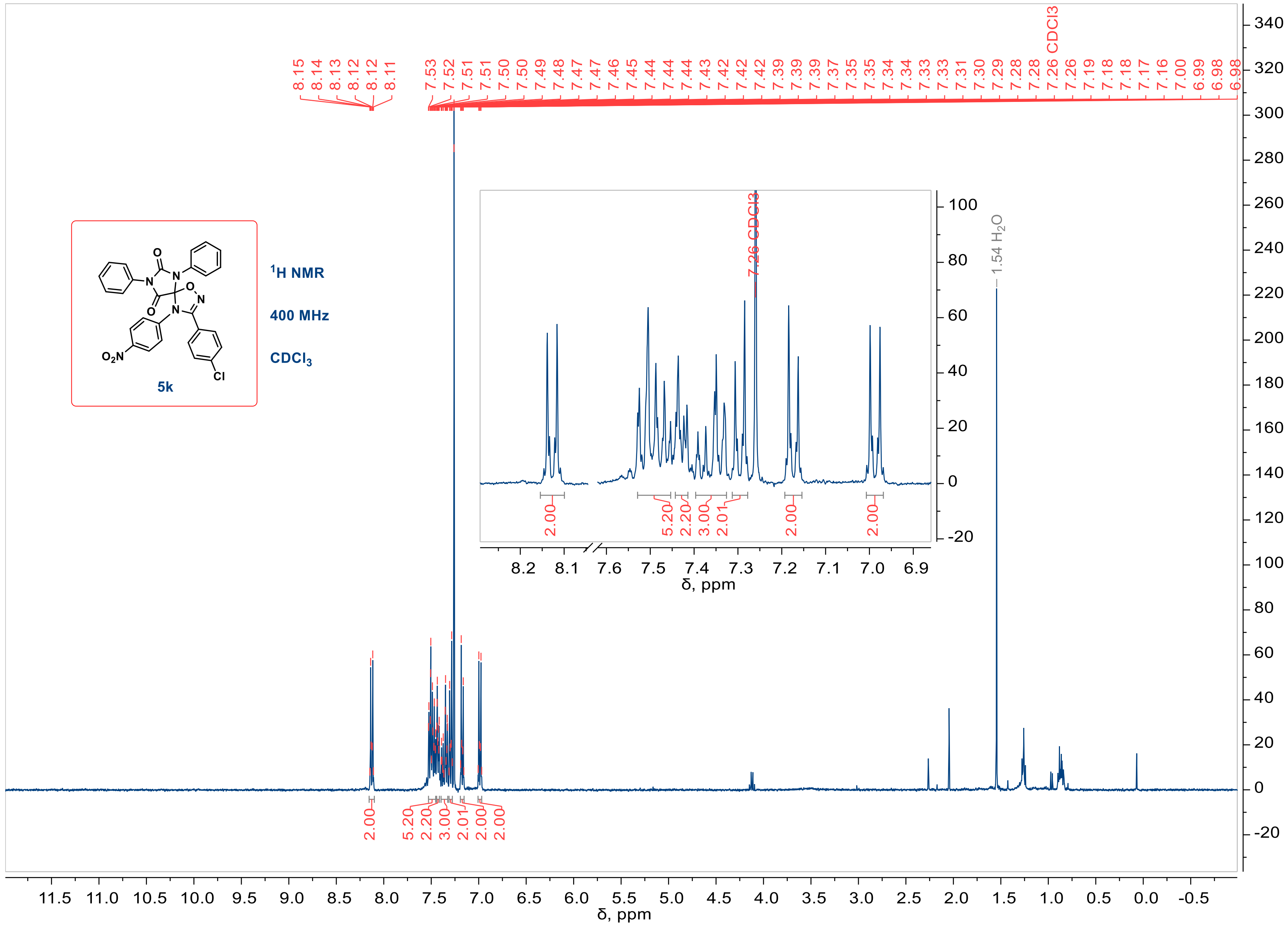

Figure S22. ^{1}H NMR spectrum of compound **5k** ($CDCl_3$, 400 MHz).

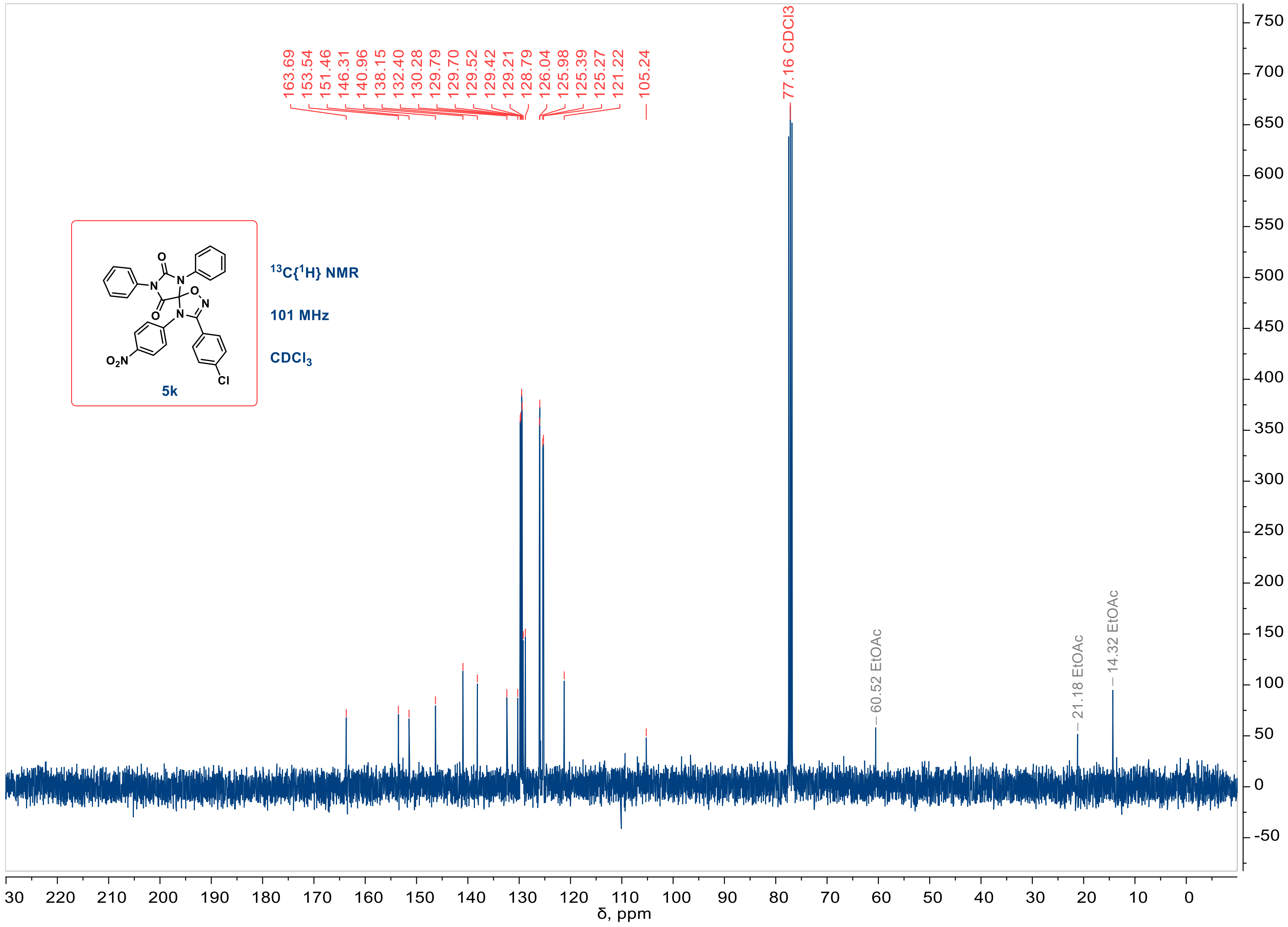

Figure S23. $^{13}C\{1H\}$ NMR spectrum of compound **5k** ($CDCl_3$, 101 MHz).

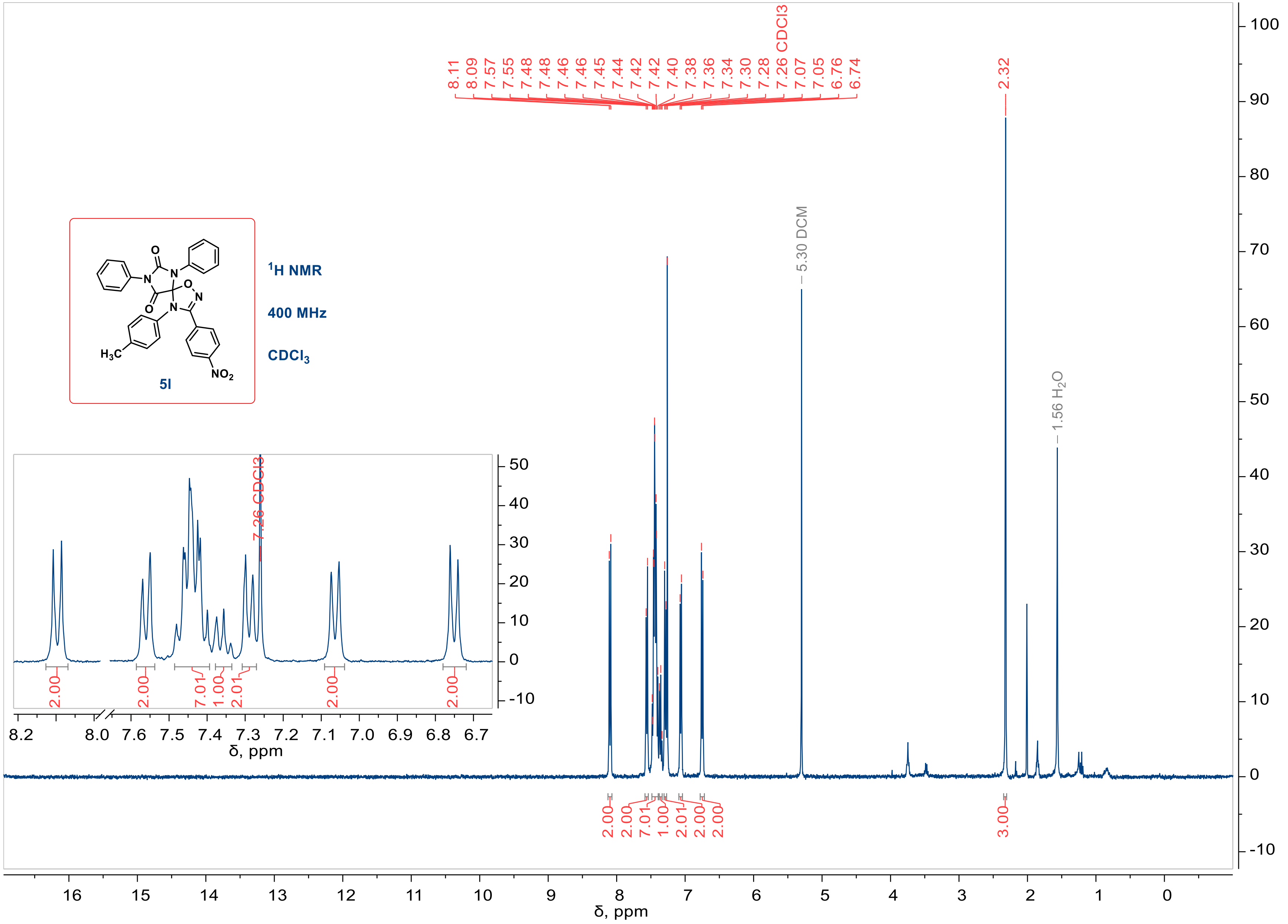

Figure S24. ^{1}H NMR spectrum of compound **5l** ($CDCl_3$, 400 MHz).

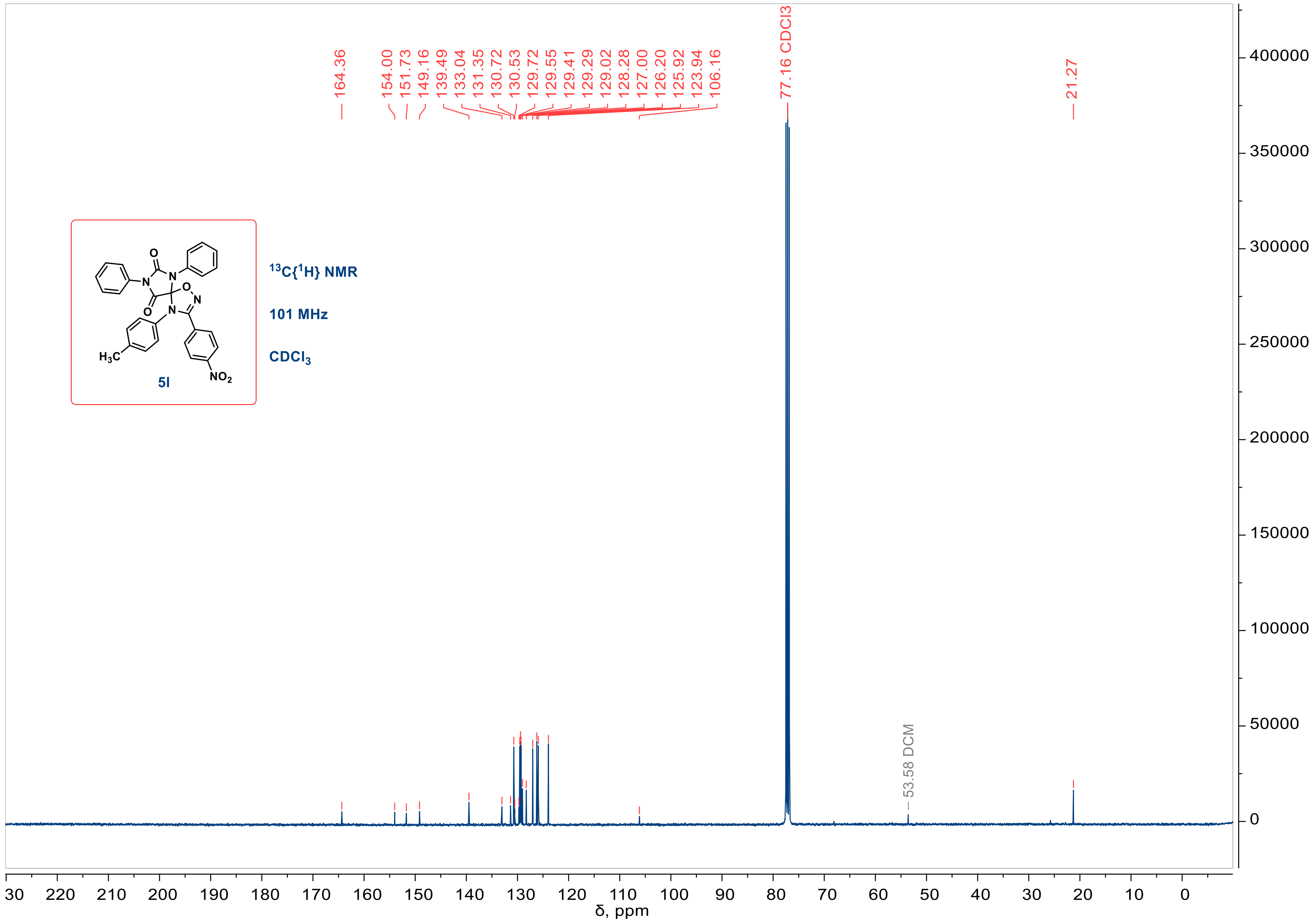

Figure S25. ^{13}C{1H} NMR spectrum of compound **5l** ($CDCl_3$, 101 MHz).
